# Sampling Period Contributes More than Cultivar to Shaping the Olive (*Olea europaea* L.) Leaf Metabolome: A Full-Season LC-MS/MS Survey

**DOI:** 10.3390/plants15213264

**Published:** 2026-10-22

**Authors:** Igor Pasković, Simone Bernobić, Dalibor Broznić, Nikola Major, Smiljana Goreta Ban, Nataly Milovan, Šime Marcelić, Ivan Dlačić, Dean Ban, Soukaina Lahmaoui, Marija Polić Pasković

**Affiliations:** 1 Department of Agriculture and Nutrition, Institute of Agriculture and Tourism, Karla Huguesa 8, 52440 Poreč, Croatia; simone@iptpo.hr (S.B.); nikola@iptpo.hr (N.M.); smilja@iptpo.hr (S.G.B.); nataly@iptpo.hr (N.M.); ivan@iptpo.hr (I.D.); dean@iptpo.hr (D.B.); soukainalahmaoui123@gmail.com (S.L.); 2 Department of Medical Chemistry, Biochemistry and Clinical Chemistry, Faculty of Medicine, University of Rijeka, Braće Branchetta 20, 51000 Rijeka, Croatia; dalibor.broznic@medri.uniri.hr; 3 Department of Environmental Health, Teaching Institute of Public Health of Primorje-Gorski Kotar County, Krešimirova 52a, 51000 Rijeka, Croatia; 4 Department for Ecology, Agronomy and Aquaculture, University of Zadar, Trg Kneza Višeslava 9, 23000 Zadar, Croatia; simemarcelic@unizd.hr

**Keywords:** by-product valorisation, cultivar, LC-MS/MS, leaf metabolome, *Olea europaea* L., oleuropein, PERMANOVA, PLS-DA, repeated-measures ANOVA, seasonal dynamics, secoiridoids

## Abstract

Olive (*Olea europaea* L.) leaf composition depends on both collection date and cultivar, yet these two sources of variation are rarely separated within one orchard over a full year. Leaves of the indigenous Istrian cultivars ‘Istarska bjelica’ and ‘Rošinjola’ and the introduced ‘Leccino’ were sampled in one orchard every two months from April 2025 to February 2026. Of 82 metabolites quantified by LC-MS/MS, 64 were detected in every cultivar and period and analysed. The sampling period significantly affected 56 metabolites, while cultivar influenced 35. Their interaction was significant for 23 metabolites, indicating that seasonal changes are cultivar-dependent. Effect sizes confirmed this ordering: PERMANOVA attributed 45.3% of the total metabolome variance to sampling period against 17.3% to cultivar. At the whole-metabolome level, sampling period affected all three cultivars, whereas cultivar differentiation was absent in one period. By class, sampling period affected all six classes, whereas cultivar was significant for phenolic acids, flavonoids and B-group vitamins, with no effect on the total metabolite pool (*p* = 0.189). Unsupervised PCA grouped samples by season, and supervised PLS-DA detected cultivar differences (96.3% cross-validated accuracy, permutation *p* = 0.012) although they were not resolved by the first two principal components; this accuracy describes separability within this dataset of nine experimental units rather than predictive performance. Cultivar information is therefore spread across many metabolites, and fixed sampling dates are essential when comparing cultivars or assessing leaves as raw material.

## 1. Introduction

Olive (*Olea europaea* L.) is a Mediterranean fruit deeply rooted in Mediterranean history, religion, economy, and lifestyle. Although it is primarily grown for olive oil and fruit, recent research has shifted more interest toward olive by-products. Therefore, the main objectives are now refocusing on better understanding the olive metabolome and developing strategies to deliver higher-value raw materials from olive groves [1,2,3,4]. In this context, olive leaves are recognised as a valuable by-product of olive cultivation, accounting for approximately 10–25% of the total biomass removed from olive trees during pruning and harvesting. Due to their rich chemical composition, particularly their high concentration of phenolic compounds as secondary metabolites, olive leaves are valued in numerous sectors, including the food and pharmaceutical industries. They are widely used in pharmaceutical and nutraceutical formulations and as natural food preservatives [5,6,7]. Beyond these established uses, recent research has explored integrating them into sustainable packaging systems to improve functional performance and extend product shelf life [6] or using them as a component of animal feed [8,9]. The biosynthesis of plant phenolic compounds, including those found in olives, is closely linked to the shikimate pathway, which provides aromatic amino acids, particularly phenylalanine and tyrosine, and to downstream phenylpropanoid metabolism [10,11]. Although its influence is less pronounced, tryptophan may also affect phenolic compound production, underscoring the interconnections of these metabolic pathways [12]. The shikimate pathway occurs in bacteria, fungi, algae, and plants but is absent in animals [13]. Consequently, the shikimate pathway and aromatic amino acid biosynthesis represent important targets for herbicides and antimicrobial agents, as this pathway is absent in animals, including humans, providing a basis for selective toxicity [14,15]. Shikimic acid is a key intermediate in the shikimate pathway, whereas quinic acid is a structurally related metabolite associated with this pathway through dehydroquinate and dehydroshikimate metabolism [16,17]. Quinic acid also participates in the formation of hydroxycinnamoylquinic acids and other phenolic compounds [18,19]. Most enzymes involved in the shikimate pathway and in the biosynthesis of tryptophan, phenylalanine, and tyrosine are located in plastids [20]. In the olive (*Olea europaea* L.), oleuropein is the major secoiridoid glycoside and plays an important role in plant defence against various stressors and in maintaining metabolic homeostasis. Composed of a hydroxytyrosol moiety, glucose, and elenolic acid, oleuropein functions as a specialised chemical defence and, for example, serves as a precursor of protein-denaturing compounds that are activated upon tissue damage to deter herbivores [12,21] or in the olive response to other biotic and abiotic stresses [22]. In addition to its defensive role, this secoiridoid is also highly valued for its pharmacological effects, as well as for its significant contribution to the quality and nutritional profile of olives and olive-derived products [23,24,25,26]. However, there are other secondary metabolites in olive leaves to which attention can be directed. Flavonoids contribute to the antioxidant capacity and the phenolic composition of the leaf. Luteolin-7-O-glucoside, apigenin-7-O-glucoside and quercetin glycosides accumulate in olive leaves under stress conditions, counteracting stress-induced ROS accumulation [10]. Flavonoids and secoiridoids also show higher cultivar-related signatures across the season [27]. In the amino acid profile of olive leaves, variations in primary metabolism are strongly linked to nitrogen metabolism dynamics, nutrient management, and seasonal changes [22,28,29,30].

Although the phenolic profiles of olive leaves are relatively well described under Mediterranean agro-ecological conditions, research linking these profiles to leaf primary metabolism remains limited, particularly regarding monitoring across the entire vegetative cycle. Therefore, this study aimed to determine how cultivar, sampling time across different stages of olive growth and development, and their interaction influence the metabolic profile of olive leaves during the vegetation period and into the next year’s growth. In three cultivars, which stood out in our previous research ‘Leccino’, ‘Istarska bjelica’, and ‘Rošinjola’—a total of 64 metabolites from six different groups were quantified across six sampling times from April 2025 to February 2026 on trees of the same habitus, health status, and age. These groups included amino acids and other nitrogen compounds, B-group vitamins, organic acids including shikimic and quinic acid, phenolic acids, flavonoids, and secoiridoids. Three hypotheses were formulated: (i) seasonal dynamics are the main source of variability for most metabolites, while cultivar differences are more pronounced in the flavonoid and secoiridoid groups; (ii) amino acids associated with the shikimate pathway follow a seasonal pattern of phenolic compounds, indicating a connection between primary and secondary metabolism in the olive leaf; and (iii) cultivar-specific responses throughout the season, i.e., the interaction between cultivar and sampling time, significantly contribute to the overall variability of the metabolome. We believe that answers to these questions will not only contribute to understanding the seasonal regulation of olive leaf metabolism in different cultivars but also confirm the period when the olive leaf has the most favourable chemical composition for use as a raw material in various industries.

## 2. Materials and Methods

The experiment was conducted in a national collection olive orchard (*Olea europaea* L.) at the Institute of Agriculture and Tourism in Poreč, Croatia, as described in our previous study [4]. The research involved three olive cultivars: the indigenous Istrian cultivars ‘Istarska bjelica’ and ‘Rošinjola’, and the introduced cultivar ‘Leccino’, one of the most economically important cultivars in newly established Croatian olive orchards. ‘Istarska bjelica’ and ‘Leccino’ are vigorous cultivars with dense canopies, partially self-fertile and regularly bearing fruit, with ‘Istarska bjelica’ showing an upright growth habit and ‘Leccino’ a characteristic drooping habit [31]. ‘Rošinjola’ is moderately vigorous, with a spreading and dense canopy and short elliptical leaves [4].

The experiment included two factors: cultivar (three levels) and sampling period (six levels), with three biological replicates per cultivar. Each replicate consisted of three trees whose leaves were pooled into a single analytical sample; the three-tree replicate, not the individual tree, was therefore the independent experimental unit. The same experimental units were sampled at all six sampling periods, which allowed temporal changes to be followed within each experimental unit while accounting for variability among experimental units. In total, the study included 27 trees (3 cultivars × 3 experimental units × 3 trees per unit), yielding 54 pooled samples (3 cultivars × 3 experimental units × 6 sampling periods).

Sampling was carried out every two months from mid-April 2025 to mid-February 2026, comprising six sampling rounds (R1–R6): mid-April (vegetative growth/pre-flowering), mid-June (fruit set), mid-August (fruit growth and early oil accumulation), mid-October (harvest), mid-December (winter dormancy), and mid-February (late winter/dormancy). All trees in the orchard followed the same regular agronomic practices (pruning, plant protection, fertilisation, and inter-row management), with no experimental treatments during the study period. Leaves were collected evenly around the canopy from the middle section of current-season branches to ensure uniformity and representative sampling across the canopy. After each sampling, leaves were gently washed in the lab, starting with tap water, then with approximately 5000 mL of 1% acetic acid in deionized water per 120 leaves (one sample), immersed and gently agitated for about 30 s, and finally rinsed twice with deionized water. The wash removes adhering soil, dust, and foliar spray residues that would otherwise bias elemental and surface analyses, and is the procedure consistently used [3,4,32]. Exposure to acetic acid is brief and identical for every sample; removal of a small fraction of cuticular or epicuticular compounds cannot be excluded, so the concentrations reported are those of the washed leaf. They were air-dried to constant weight and ground into a fine powder using a Retsch ZM 200 mill (Retsch GmbH, Haan, Germany) before analysis [32].

### 2.1. Chemicals and Standards

All solvents used were of HPLC grade. Methanol and acetonitrile were sourced from Merck (Darmstadt, Germany), while phosphoric acid came from Sigma-Aldrich (St. Louis, MO, USA). The phenolic compounds’ analytical standards had a purity of ≥ 98.0% (Extrasynthese, Genay, France). Deionised water was produced with a Siemens UltraClear system (Siemens AG, München, Germany). The chemicals and standards matched those used in our earlier research on the same orchard [3,4].

### 2.2. Determination of Primary and Secondary Metabolites

Extraction was carried out as previously described [3]. Ground olive leaves (30 mg) were suspended in 1.5 mL of 80% aqueous methanol and sonicated in a bath (Sonorex Digitec; Bandelin electronic, Berlin, Germany) for 15 min. The supernatants were separated by centrifugation at 5000 rpm for 5 min (Domel Centric 350, Železniki, Slovenia) and filtered through 0.20 µm cellulose filters. Each filtrate was stored refrigerated at 4 °C in the dark until analysis.

Primary metabolites were analysed using the same LC-MS/MS platform with the method from Polić Pasković et al. [3], on a Discovery HS F 5- 3 column (2.1 mm × 150 mm, 3 µm core–shell; Sigma-Aldrich, St. Louis, MO, USA) at 37 °C. The injection volume was 1 µL, with mobile phases A (water with 0.1% formic acid) and B (acetonitrile with 0.1% formic acid) at 0.25 mL/min. The gradient was: 0–2 min, 100% A; 2–5 min, from 100% A to 75% A; 5–11 min, from 75% A to 65% A; 11–15 min, from 65% A to 5% A; 15–20 min, 5% A; 20–20.1 min, from 5% A to 100% A; and 20.1–25 min, 100% A.

Secondary metabolites were quantified using LC-MS/MS following the protocol used in the same orchard by Žurga et al. [4]. The system included a Nexera HPLC 40 XS and an LCMS-8045 triple quadrupole mass spectrometer (Shimadzu, Kyoto, Japan). Separation was performed on a C 18 core–shell column (2.1 mm × 150 mm, 2.7 µm; Agilent Technologies, Palo Alto, CA, USA) at 37 °C, with an injection volume of 1 µL. A linear gradient of mobile phase A (water with 0.1% acetic acid) and mobile phase B (methanol with 0.1% acetic acid) was applied at 0.35 mL/min as follows: 0–0.75 min, 98% A; 0.75–15 min, from 98% A to 50% A; 15–15.1 min, from 50% A to 0% A; 15.1–20 min, 0% A; 20–20.1 min, from 0% A to 98% A; and 20.1–25 min, 98% A.

For both analyses, metabolites were identified and quantified by matching retention times, ion transitions, and peak areas with standards. Blanks were prepared and analysed the same way; only calibration curves with R^2^> 0.99 were used for concentration calculations [3]. Calibration ranges, limits of detection and quantification and the quantifier and qualifier MRM transitions with their ion-ratio acceptance criteria for all 82 quantified metabolites are given in Supplementary Table S1, together with the completeness of each; these limits refer to concentrations in the injected extract. Internal standards were not used; quantification relied on external calibration with authentic standards for each analyte. All samples were extracted and analysed within a single batch under identical conditions, and values are reported as measured, without correction. Analysis in a single batch removes inter-batch drift but does not compensate for sample-specific variation in extraction recovery, matrix effects or ion suppression/enhancement, so absolute concentrations should be regarded as semi-quantitative; the cultivar and sampling-period comparisons rest on relative differences measured under identical conditions and are unaffected by a response factor common to all samples. For 15 of the 64 metabolites, a proportion of values fell between the limits of detection and quantification; these were retained and are reported as measured. Results are expressed as mg 100 g^−1^ dry weight (DW).

### 2.3. Statistical Analysis

Data completeness was evaluated for each quantified metabolite before inferential analysis. Of the 82 quantified metabolites, 64 showed complete measurements across all cultivars and sampling periods (valid n = 54) and were retained for analysis. The remaining 18 had at least one value below the detection or quantification limits and were omitted, because the repeated-measures design requires a complete series for every experimental unit: a single missing observation removes that unit from the model. Their completeness ranged from 11% to 98% and is listed in Supplementary Table S1 together with the limits of quantification for all analysed metabolites. The same 64 metabolites were used for all analyses. The dataset comprised three olive cultivars (‘Leccino’, ‘Rošinjola’ and ‘Istarska bjelica’) with three pooled samples per cultivar, each sampled at all six sampling periods (April, June, August, October and December 2025, and February 2026), giving 9 experimental units and 54 samples. Repeated-measures analysis of variance was applied separately to each of the 64 metabolites, with cultivar as a between-subject factor and sampling period as a within-subject factor; the interaction tested whether temporal metabolite changes differed among cultivars. Concentrations were analysed on the raw scale. Sphericity was tested with Mauchly’s test, which was violated for most metabolites (44 of 64 metabolites; median Greenhouse–Geisser ε = 0.36), and the Greenhouse–Geisser correction was therefore applied to all within-subject effects since Mauchly’s test is underpowered at this sample size and the correction is inconsequential where sphericity holds and applying it throughout keeps the error degrees of freedom comparable across the 64 metabolites. Significance was set at *p* ≤ 0.05. Effect sizes are reported as partial eta-squared and, so that between-subject and within-subject effects could be compared on a common denominator, as generalised eta-squared. Significant effects prompted pairwise comparisons. Cultivar means were compared by Tukey’s HSD, which is exact for a between-subject factor with equal replication. Sampling-period and interaction means were compared by pairwise *t*-tests on the within-subject error term with Bonferroni adjustment, since the sphericity assumption required by Tukey’s test was not met within subjects. For metabolites with a significant cultivar × sampling period interaction, main effects were not interpreted separately and simple effects were tested instead. Cultivars were compared within each sampling period using Tukey-adjusted comparisons, and sampling periods within each cultivar using Bonferroni-adjusted pairwise tests. Cell means with their standard errors and post hoc letters for all 64 metabolites are tabulated for each factor level in Supplementary Tables S2 and S3, and letter groupings for all significant interactions in Supplementary Table S4. Homogeneity of variances was checked with Brown–Forsythe tests and residual normality with Shapiro–Wilk tests, with per-metabolite values given in Supplementary Table S5. Variances were homogeneous between cultivars for all 64 metabolites but differed between sampling periods for 30 of them; that heterogeneity is absorbed by the sphericity correction, because the within-subject assumption is sphericity rather than equality of variances. Friedman tests confirmed the sampling-period decision for 58 of the 64 metabolites. The Benjamini–Hochberg correction was applied post hoc with the family defined in two ways, within each model term (64 tests per term) and within each compound (3 tests per compound). The two outcomes are not combined into a single decision rule, and the counts retained under each definition are reported separately. Of 114 effects significant at raw *p* ≤ 0.05, 94 were retained under both definitions and 3 under neither; the latter are marked in corresponding tables. The remaining 17 were retained under one definition only and are therefore scheme-dependent; they are significant but unmarked in Table 1, Table 2, Table 3, Table 4, Table 5 and Table 6. Adjusted values for all 192 tests are given in Supplementary Table S5.

All multivariate analyses were run on the same matrix: log-transformed, *z*-standardised concentrations of the 64 metabolites (n = 54). Principal component (PCA) analysis was computed on the correlation matrix, and scores were examined by cultivar and sampling period, with factor loadings given in Supplementary Table S6. Permutational multivariate analysis of variance (PERMANOVA) [33,34] was applied to the Euclidean distance matrix and fitted sequentially by terms (sampling period, cultivar and their interaction), with variance explained expressed as *R*^2^. Because the design is fully balanced—three cultivars × six sampling periods × three experimental units, with no missing samples—the model terms are orthogonal, and the sequential sums of squares do not depend on the order in which terms are entered; fitting cultivar before sampling period returns the same variance components. Homogeneity of multivariate dispersions was verified with PERMDISP [35] on the same distances. Permutation was constrained to the split-plot structure of the design [36]: sampling-period labels were permuted within experimental units and cultivar labels between whole units. PERMANOVA used 999 constrained permutations and PERMDISP 999 unrestricted ones. Partial least squares discriminant analysis (PLS-DA) used one-hot encoded class membership and was cross-validated by leaving out one experimental unit at a time, so that all six samples of a unit were excluded together. Eight components were retained for sampling period and four for cultivar, chosen from the cross-validation results. Model significance was assessed by permutation testing, with sampling-period labels permuted within experimental units and cultivar labels between whole units (999 and 249 permutations, respectively). Metabolites with a variable importance in projection (VIP) score above 1 [37] were taken as important for group separation; VIP was computed separately for the cultivar and sampling-period models. Spearman’s rank correlations among metabolites were computed on the untransformed concentrations, rank correlation being invariant to the log transformation; pairs with |ρ| ≥ 0.70 are listed in Supplementary Table S7 and are exploratory, since the same units were sampled repeatedly.

All statistical analyses were carried out in R 4.4.1 (R Core Team, Vienna, Austria). Data were imported with readxl 1.4.3. The repeated-measures analyses of variance, Mauchly’s test, the Greenhouse–Geisser correction, Brown–Forsythe and Shapiro–Wilk tests and the Friedman tests were computed with the base stats package, and tabular output was written with openxlsx 4.2.7.1. Principal component analysis and the distance matrices were computed with the base stats package, PERMDISP with vegan 2.6-8 (betadisper and permutest), PLS-DA and VIP scores with pls 2.8.5, and the generalised inverse used in the sequential PERMANOVA with MASS 7.3-60.2. Figures were produced with ggplot2 3.5.1, cowplot 1.1.3 and scales 1.3.0. Preliminary analyses and independent verification of the results obtained using the R code were performed with TIBCO Statistica 14.1.0.8 (TIBCO StatSoft^®^, Palo Alto, CA, USA).

## 3. Results

### 3.1. Amino Acids and Other Nitrogen-Containing Compounds

Sampling period had a pronounced effect on the amino acid profile, with significant temporal variation observed for all 22 amino acids analysed (Table 1). Cultivar significantly affected 11 compounds, while cultivar × sampling period interactions were significant for 10. The strongest temporal effects were observed for glutamine (ηp^2^ = 0.935), 4-aminobutyric acid (GABA; ηp^2^ = 0.924), glutamic acid (ηp^2^ = 0.922), and leucine (ηp^2^ = 0.915). In contrast, threonine, valine, isoleucine, leucine, and 2-aminobutyric acid varied significantly across sampling periods without significant cultivar or interaction effects, indicating broadly similar temporal patterns among the three cultivars. Among the other nitrogen-containing compounds, choline and dimethylglycine were affected by sampling period (both *p* < 0.001), whereas neither cultivar nor the cultivar × sampling period interaction was significant.

**Table 1:** Effects of cultivar, sampling period, and their interaction on amino acids and other nitrogen-containing compounds in olive samples.

Compound	Cultivar *p* (ηp^2^)	Sampling Period *p* ^†^ (ηp^2^)	Cultivar × Sampling Period *p* ^†^ (ηp^2^)
aspartic acid	0.160 (0.457)	**0.001 (0.708)**	0.307 (0.313)
asparagine	**0.040 (0.657)** ^‡^	**<0.001 (0.798)**	0.177 (0.414)
serine	0.309 (0.324)	**0.003 (0.777)**	0.551 (0.186)
glycine	**0.001 (0.897)**	**0.012 (0.498)**	**0.003 (0.687)**
glutamine	**0.013 (0.763)**	**<0.001 (0.935)**	**0.038 (0.552)**
threonine	0.400 (0.263)	**0.001 (0.814)**	0.521 (0.201)
alanine	**0.100 (0.536)**	**<0.001 (0.820)**	0.329 (0.303)
glutamic acid	**0.136 (0.485)**	**<0.001 (0.922)**	**0.019 (0.563)**
methionine sulfoxide	**0.059 (0.611)**	**<0.001 (0.911)**	0.200 (0.389)
citrulline	**0.068 (0.591)**	**<0.001 (0.829)**	**0.063 (0.544)**
proline	**0.019 (0.734)**	**0.004 (0.680)**	**0.148 (0.433)**
valine	0.725 (0.102)	**<0.001 (0.886)**	0.418 (0.254)
histidine	**0.005 (0.825)**	**0.004 (0.675)**	**0.037 (0.597)**
arginine	**<0.001 (0.943)**	**<0.001 (0.870)**	**0.001 (0.812)**
4-aminobutyric acid (GABA)	**<0.001 (0.908)**	**<0.001 (0.924)**	**<0.001 (0.870)**
isoleucine	0.347 (0.297)	**<0.001 (0.893)**	0.206 (0.405)
tyrosine	**<0.001 (0.906)**	**<0.001 (0.814)**	**0.001 (0.709)**
leucine	0.178 (0.438)	**<0.001 (0.915)**	0.073 (0.573)
phenylalanine	**<0.001 (0.919)**	**<0.001 (0.799)**	**0.028 (0.594)**
tryptophan	**<0.001 (0.907)**	**<0.001 (0.859)**	**0.034 (0.605)**
choline	0.918 (0.028)	**<0.001 (0.715)**	0.168 (0.392)
dimethylglycine	1.000 (0.000)	**<0.001 (0.756)**	0.284 (0.322)
lysine	**<0.001 (0.920)**	**0.001 (0.718)**	**0.013 (0.665)**
2-Aminobutyric acid	0.787 (0.077)	**0.002 (0.795)**	0.405 (0.262)

^†^ For within-subject effects, Greenhouse–Geisser-corrected *p*-values were reported throughout; the between-subject effect of cultivar needs no such correction. The *p*-values shown are raw; Benjamini–Hochberg-adjusted values are given in Supplementary Table S5. Values in parentheses represent partial eta-squared (ηp^2^). Statistically significant effects (*p* ≤ 0.05) are shown in bold; ^‡^ raw *p* ≤ 0.05 but not retained under either Benjamini–Hochberg definition.

**Figure 1:**
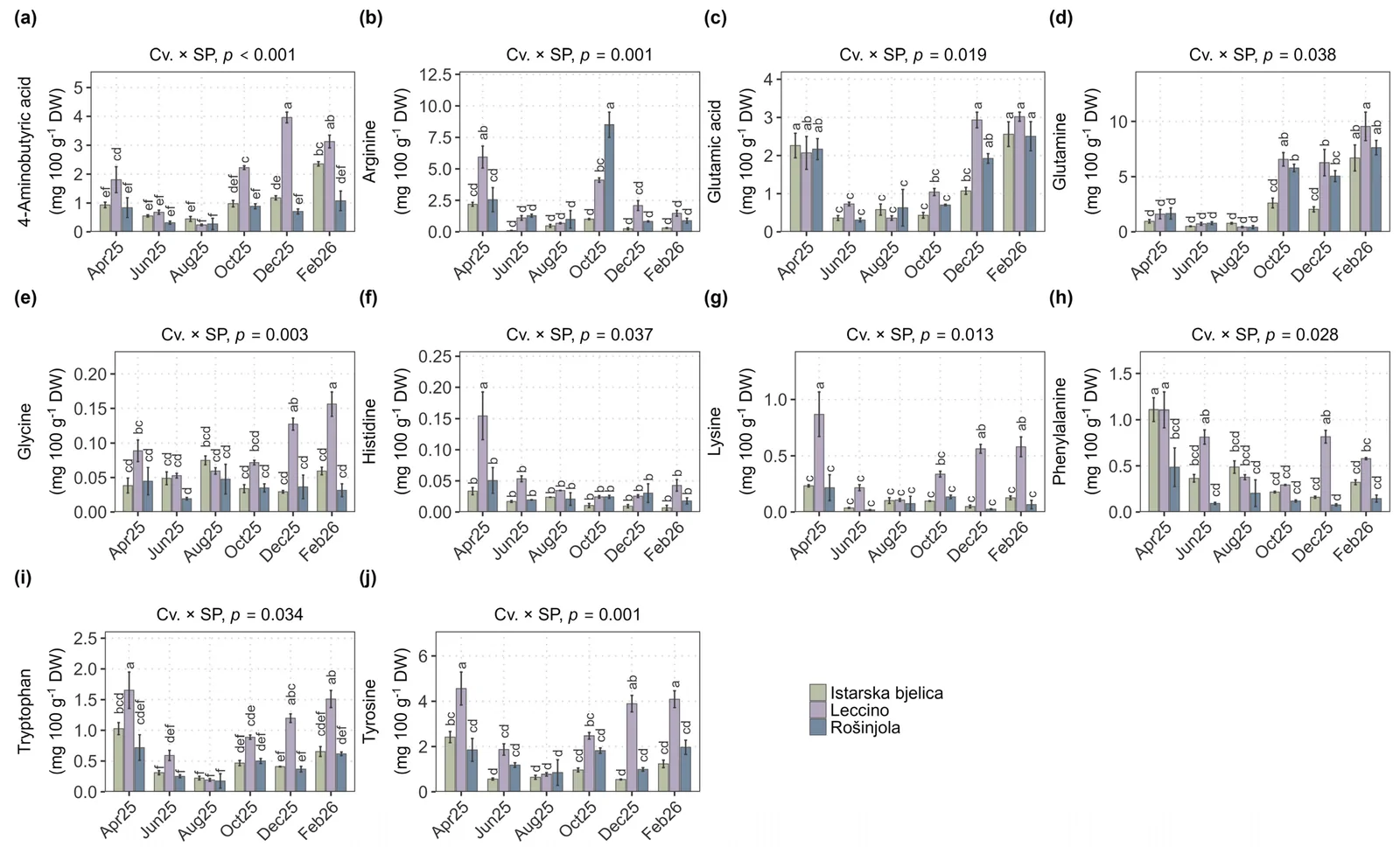
Temporal changes in amino acids and other nitrogen-containing compounds showing a significant cultivar × sampling period interaction, among three olive cultivars from April 2025 to February 2026. (**a**) 4-Aminobutyric acid (GABA), (**b**) arginine, (**c**) glutamic acid, (**d**) glutamine, (**e**) glycine, (**f)** histidine, (**g**) lysine, (**h**) phenylalanine, (**i**) tryptophan, (**j**) tyrosine. Values are presented as means ± standard errors (n = 3). For significant cultivar × sampling period interactions, cultivars were compared within sampling periods using Tukey’s test and sampling periods within cultivars using Bonferroni-adjusted comparisons (*p* ≤ 0.05); different letters indicate significant simple-effect differences. The *p*-value above each panel is the Greenhouse–Geisser-corrected interaction term.

Cultivar-dependent temporal variation was most pronounced for arginine and GABA, with interaction effect sizes of ηp^2^ = 0.812 and 0.870, respectively (Table 1). Arginine concentrations differed markedly among cultivars in October, when the increase was particularly pronounced in ‘Rošinjola’ (Figure 1b). GABA concentrations declined during the earlier sampling periods in all three cultivars, followed by a marked increase in ‘Leccino’ later in the study (Figure 1a). Tyrosine also showed a strong cultivar effect (ηp^2^ = 0.906) and a significant cultivar × sampling period interaction (ηp^2^ = 0.709). After reaching relatively low concentrations in August, tyrosine increased substantially in ‘Leccino’, whereas the increase was less pronounced in ‘Rošinjola’ and ‘Istarska bjelica’ (Figure 1j). Significant cultivar × sampling period interactions were additionally found for glutamic acid, glutamine, glycine, histidine, lysine, phenylalanine and tryptophan, all of which are shown in Figure 1c–i; the interaction for citrulline did not reach significance after Greenhouse–Geisser correction (*p* = 0.063).

### 3.2. B-Group Vitamins and Vitamers

In addition to the changes observed in amino acids and other nitrogen-containing compounds, B-group vitamins and vitamers also varied across the sampling period (Table 2). Significant temporal effects were observed for three of the four compounds, although their magnitude differed considerably. The strongest effect was found for niacinamide (ηp^2^ = 0.892), followed by riboflavin (ηp^2^ = 0.630) and nicotinic acid (ηp^2^ = 0.563), whereas pyridoxine showed no significant temporal variation (*p* = 0.383). Cultivar significantly affected two of the four compounds, riboflavin (ηp^2^ = 0.786) and pyridoxine (ηp^2^ = 0.773).

No significant cultivar × sampling period interactions were detected in this group, although nicotinic acid (*p* = 0.065) and riboflavin (*p* = 0.101) approached significance. Accordingly, no figure is presented for this compound group; cultivar and sampling period main effects are reported in Table 2.

**Table 2:** Effects of cultivar, sampling period, and their interaction on B-group vitamins and vitamers in olive samples.

Compound	Cultivar *p* (ηp^2^)	Sampling Period *p* ^†^ (ηp^2^)	Cultivar × Sampling Period *p* ^†^ (ηp^2^)
nicotinic acid (niacin)	**0.079 (0.571)**	**0.002 (0.563)**	**0.054 (0.466)**
niacinamide (nicotinamide)	**0.066 (0.596)**	**<0.001 (0.892)**	0.724 (0.155)
riboflavin (vitamin B2)	**0.010 (0.786)**	**<0.001 (0.630)**	**0.102 (0.419)**
pyridoxine	**0.012 (0.773)**	**0.378 (0.142)**	0.875 (0.063)

^†^ For within-subject effects, Greenhouse–Geisser-corrected *p*-values were reported throughout; the between-subject effect of cultivar needs no such correction. The *p*-values shown are raw; Benjamini–Hochberg-adjusted values are given in Supplementary Table S5. Values in parentheses represent partial eta-squared (ηp^2^). Statistically significant effects (*p* ≤ 0.05) are shown in bold.

### 3.3. Organic Acids and Related Metabolites

Organic acids and related metabolites were strongly influenced by cultivar and sampling period (Table 3). Significant cultivar effects were observed for four of the seven compounds, one of them (quinic acid) only at the uncorrected level, with the largest effects for shikimic acid (ηp^2^ = 0.904) and malic acid (ηp^2^ = 0.801). Sampling period significantly affected quinic, malic, shikimic, and succinic acids, with the strongest effects observed for quinic acid (ηp^2^ = 0.916), shikimic acid (ηp^2^ = 0.858), and malic acid (ηp^2^ = 0.835). Citric acid and ascorbic acid showed no significant temporal effect. Jasmonic acid was significantly affected by cultivar (*p* = 0.019) but not by sampling period (*p* = 0.090), and their interaction was not significant.

**Figure 2:**
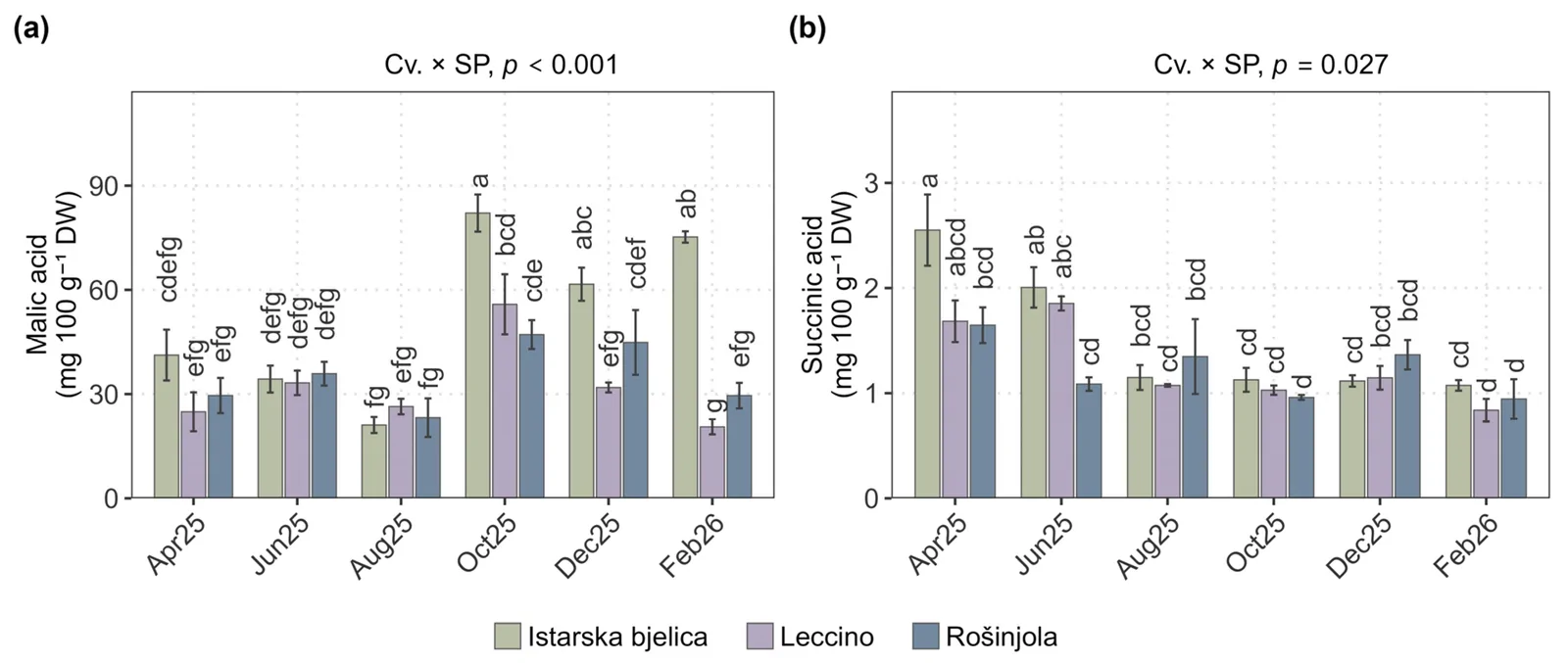
Temporal changes in organic acids showing a significant cultivar × sampling period interaction, among three olive cultivars from April 2025 to February 2026. (**a**) Malic acid, (**b**) succinic acid. Values are presented as means ± standard errors (n = 3). For significant cultivar × sampling period interactions, cultivars were compared within sampling periods using Tukey’s test and sampling periods within cultivars using Bonferroni-adjusted comparisons (*p* ≤ 0.05); different letters indicate significant simple-effect differences. The *p*-value above each panel is the Greenhouse–Geisser-corrected interaction term.

Significant cultivar × sampling period interactions were observed for malic acid (*p* = 0.001, ηp^2^ = 0.744) and succinic acid (*p* = 0.022, ηp^2^ = 0.554), whereas the interaction for shikimic acid did not reach significance (*p* = 0.053). Malic acid concentrations initially decreased and subsequently increased markedly in October in all three cultivars, with the highest increase observed in ‘Istarska bjelica’ (Figure 2a). Thereafter, concentrations remained comparatively high in ‘Istarska bjelica’ but decreased markedly in ‘Leccino’. Shikimic acid followed a different temporal pattern, increasing during the earlier sampling periods in ‘Leccino’ and ‘Istarska bjelica’ before subsequently declining. Succinic acid decreased steadily from April onward in ‘Istarska bjelica’, whereas the decline was less pronounced in the other two cultivars (Figure 2b). For shikimic acid the interaction did not reach significance after Greenhouse–Geisser correction (*p* = 0.053).

**Table 3:** Effects of cultivar, sampling period, and their interaction on organic acids and related metabolites in olive samples.

Compound	Cultivar *p* (ηp^2^)	Sampling Period *p* ^†^ (ηp^2^)	Cultivar × Sampling Period *p* ^†^ (ηp^2^)
quinic acid	**0.047 (0.638)** ^‡^	**<0.001 (0.916)**	0.080 (0.456)
citric acid	**0.120 (0.507)**	0.856 (0.036)	0.341 (0.291)
malic acid	**0.008 (0.801)**	**<0.001 (0.835)**	**<0.001 (0.744)**
shikimic acid	**<0.001 (0.904)**	**<0.001 (0.858)**	**0.053 (0.478)**
succinic acid	**0.109 (0.523)**	**<0.001 (0.767)**	**0.022 (0.554)**
jasmonic acid	**0.019 (0.734)**	**0.100 (0.308)**	0.818 (0.121)
ascorbic acid	0.062 (0.603)	0.068 (0.337)	0.399 (0.269)

^†^ For within-subject effects, Greenhouse–Geisser-corrected *p*-values were reported throughout; the between-subject effect of cultivar needs no such correction. The *p*-values shown are raw; Benjamini–Hochberg-adjusted values are given in Supplementary Table S5. Values in parentheses represent partial eta-squared (ηp^2^). Statistically significant effects (*p* ≤ 0.05) are shown in bold; ^‡^ raw *p* ≤ 0.05 but not retained under either Benjamini–Hochberg definition.

### 3.4. Phenolic Acids and Related Phenolic Compounds

The effects of cultivar and sampling period were less uniform among phenolic acids and related phenolic compounds than in the preceding metabolite groups (Table 4). Seven of the nine compounds varied significantly across sampling periods, with the largest temporal effects observed for 3,4-dihydroxybenzoic acid (ηp^2^ = 0.791), chlorogenic acid (ηp^2^ = 0.735), and vanillic acid-4-O-glucoside (ηp^2^ = 0.714). Cultivar significantly affected four compounds, most notably chlorogenic acid (ηp^2^ = 0.988) and 4-hydroxycinnamic acid (p-coumaric acid; ηp^2^ = 0.876). Caffeic acid and vanillin were the only compounds unaffected by cultivar, sampling period, or their interaction.

**Figure 3:**
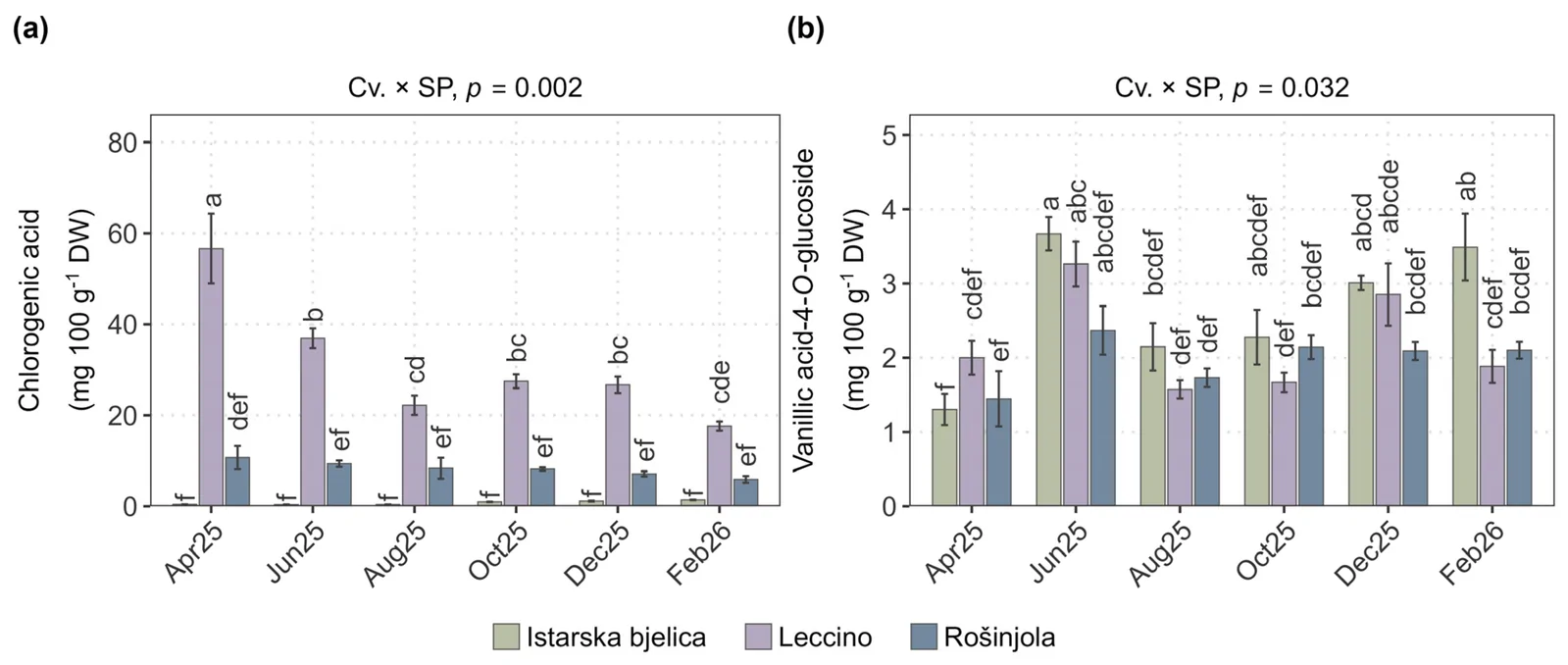
Temporal changes in phenolic acids and related phenolic compounds showing a significant cultivar × sampling period interaction, among three olive cultivars from April 2025 to February 2026. (**a**) Chlorogenic acid, (**b**) vanillic acid-4-O-glucoside. Values are presented as means ± standard errors (n = 3). For significant cultivar × sampling period interactions, cultivars were compared within sampling periods using Tukey’s test and sampling periods within cultivars using Bonferroni-adjusted comparisons (*p* ≤ 0.05); different letters indicate significant simple-effect differences. The *p*-value above each panel is the Greenhouse–Geisser-corrected interaction term.

Cultivar-dependent temporal patterns were evident for chlorogenic acid (*p* = 0.005, ηp^2^ = 0.814) and vanillic acid-4-O-glucoside (*p* = 0.028, ηp^2^ = 0.524). Among these compounds, chlorogenic acid showed the clearest cultivar differentiation, with substantially higher concentrations in ‘Leccino’ throughout the study and marked temporal variation within this cultivar (Figure 3a). Vanillic acid-4-O-glucoside followed a different pattern, with the relative differences among cultivars changing over time and becoming particularly evident in February 2026 (Figure 3b). In contrast, p-coumaric acid showed no significant cultivar × sampling period interaction (*p* = 0.454).

**Table 4:** Effects of cultivar, sampling period, and their interaction on phenolic acids and related phenolic compounds in olive samples.

Compound	Cultivar *p* (ηp^2^)	Sampling Period *p* ^†^ (ηp^2^)	Cultivar × Sampling Period *p* ^†^ (ηp^2^)
3,4-dihydroxybenzoic acid (protocatechuic acid)	**0.031 (0.687)**	**<0.001 (0.791)**	0.593 (0.185)
hydroxytyrosol	0.180 (0.435)	**0.002 (0.640)**	0.280 (0.323)
vanillic acid-4-O-glucoside	**0.025 (0.708)**	**<0.001 (0.714)**	**0.032 (0.524)**
4-hydroxybenzoic acid	0.550 (0.181)	**0.001 (0.633)**	0.087 (0.453)
chlorogenic acid	**<0.001 (0.988)**	**0.002 (0.735)**	**0.002 (0.814)**
caffeic acid	0.475 (0.220)	0.354 (0.145)	0.407 (0.259)
vanillin	0.775 (0.082)	**0.172 (0.241)**	0.266 (0.322)
4-hydroxycinnamic acid (p-coumaric acid)	**0.002 (0.876)**	**<0.001 (0.682)**	**0.452 (0.249)**
ferulic acid	**0.206 (0.409)**	**0.005 (0.700)**	0.367 (0.285)

^†^ For within-subject effects, Greenhouse–Geisser-corrected *p*-values were reported throughout; the between-subject effect of cultivar needs no such correction. The *p*-values shown are raw; Benjamini–Hochberg-adjusted values are given in Supplementary Table S5. Values in parentheses represent partial eta-squared (ηp^2^). Statistically significant effects (*p* ≤ 0.05) are shown in bold.

### 3.5. Flavonoids and Flavonoid Glycosides

Flavonoids and flavonoid glycosides exhibited marked variation in relation to both cultivar and sampling period (Table 5). Cultivar significantly affected 11 of the 16 compounds, with the largest effects observed for luteolin-7-O-glucoside (ηp^2^ = 0.962), apigenin-7-O-rutinoside (isorhoifolin; ηp^2^ = 0.943), and apigenin-7-O-glucoside (ηp^2^ = 0.942). Temporal effects were significant for 14 compounds and were strongest for naringenin-7-O-glucoside (ηp^2^ = 0.924), apigenin-7-O-rutinoside (isorhoifolin; ηp^2^ = 0.916), and luteolin-3,7-O-glucoside (ηp^2^ = 0.907).

**Figure 4:**
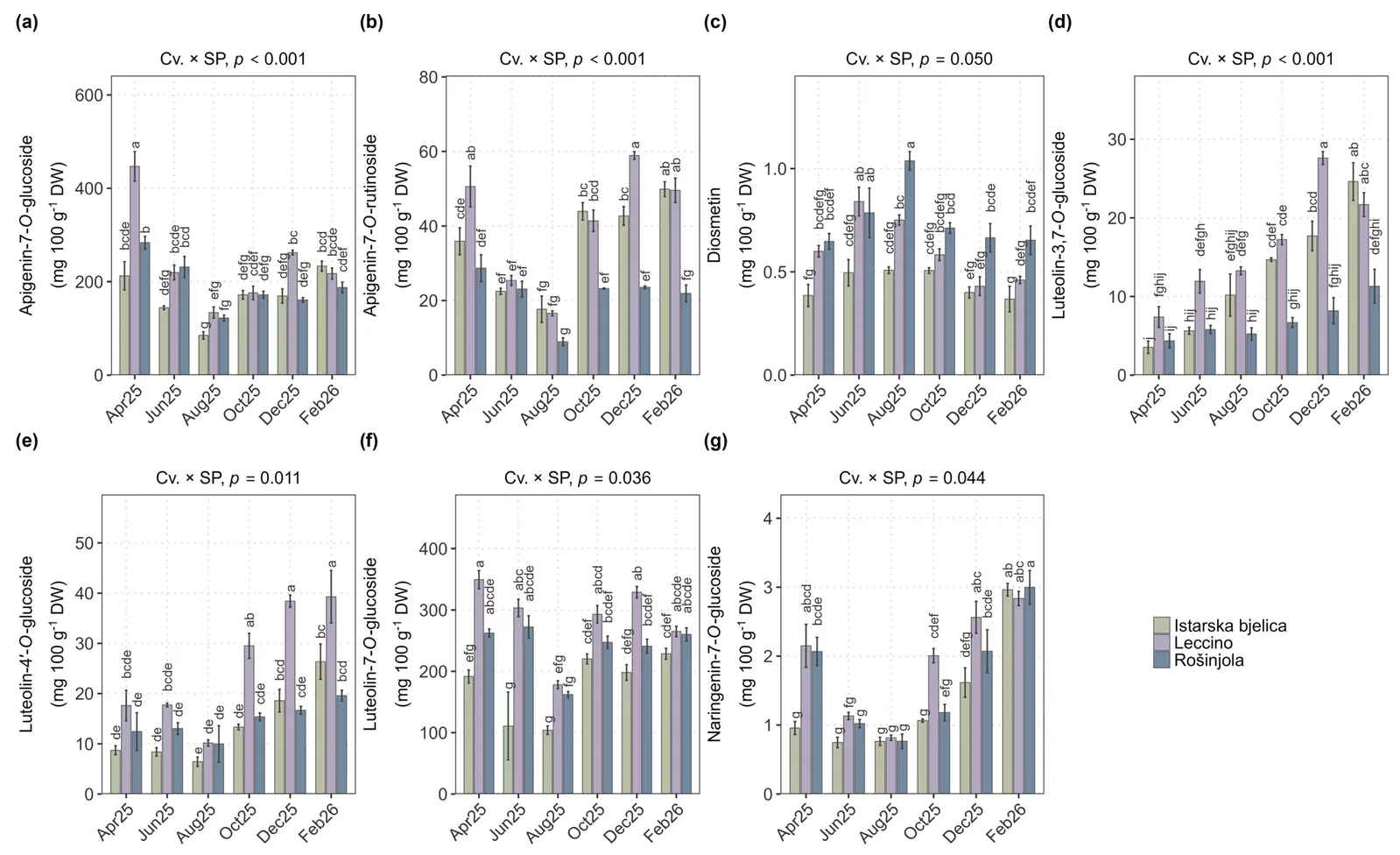
Temporal changes in flavonoids and flavonoid glycosides showing a significant cultivar × sampling period interaction, among three olive cultivars from April 2025 to February 2026. (**a**) Apigenin-7-O-glucoside, (**b**) apigenin-7-O-rutinoside (isorhoifolin), (**c**) diosmetin, (**d**) luteolin-3,7-O-glucoside, (**e**) luteolin-4′-O-glucoside, (**f**) luteolin-7-O-glucoside, (**g**) naringenin-7-O-glucoside. Values are presented as means ± standard errors (n = 3). For significant cultivar × sampling period interactions, cultivars were compared within sampling periods using Tukey’s test and sampling periods within cultivars using Bonferroni-adjusted comparisons (*p* ≤ 0.05); different letters indicate significant simple-effect differences. The *p*-value above each panel is the Greenhouse–Geisser-corrected interaction term.

Significant cultivar × sampling period interactions were identified for seven flavonoids, indicating that their temporal profiles differed among cultivars. Apigenin-7-O-glucoside showed the strongest interaction (*p* < 0.001, ηp^2^ = 0.777), with pronounced differences among cultivars that were particularly evident in April (Figure 4a). Apigenin-7-O-rutinoside (isorhoifolin) also showed a strong interaction (*p* < 0.001, ηp^2^ = 0.769), with ‘Rošinjola’ displaying a distinct profile from August onward (Figure 4b). Significant interactions were also found for diosmetin, luteolin-3,7-O-glucoside, luteolin-4′-O-glucoside, luteolin-7-O-glucoside and naringenin-7-O-glucoside, which are shown in Figure 4 (panels c–g); for apigenin, the interaction did not reach significance after Greenhouse–Geisser correction (*p* = 0.058).

**Table 5:** Effects of cultivar, sampling period, and their interaction on flavonoids and flavonoid glycosides in olive samples.

Compound	Cultivar *p* (ηp^2^)	Sampling Period *p* ^†^ (ηp^2^)	Cultivar × Sampling Period *p* ^†^ (ηp^2^)
luteolin	**<0.001 (0.936)**	**<0.001 (0.757)**	**0.220 (0.358)**
apigenin	**0.002 (0.873)**	**<0.001 (0.846)**	0.058 (0.508)
dihydroquercetin (taxifolin)	0.635 (0.140)	**<0.001 (0.793)**	0.403 (0.266)
luteolin-3,7-O-glucoside	**<0.001 (0.934)**	**<0.001 (0.907)**	**<0.001 (0.733)**
apigenin-7-O-glucoside	**<0.001 (0.942)**	**<0.001 (0.898)**	**<0.001 (0.777)**
luteolin-7-O-glucoside	**<0.001 (0.962)**	**<0.001 (0.770)**	**0.036 (0.582)**
naringenin-7-O-glucoside	**0.004 (0.842)**	**<0.001 (0.924)**	**0.044 (0.562)**
quercetin-3-*O*-rutinoside (rutin)	**0.001 (0.901)**	**0.017 (0.572)**	**0.092 (0.506)**
quercetin-3-O-glucoside (isoquercitroside)	0.649 (0.134)	**<0.001 (0.787)**	**0.150 (0.433)**
quercetin-4′-O-glucoside (spiraeoside)	0.480 (0.217)	**0.016 (0.629)**	0.441 (0.241)
apigenin-7-O-neohesperidoside (rhoifolin)	0.112 (0.519)	0.202 (0.238)	0.403 (0.267)
quercetin-3-O-rhamnoside (quercitrin)	**<0.001 (0.942)**	0.439 (0.120)	0.329 (0.304)
luteolin-4′-O-glucoside	**0.001 (0.894)**	**<0.001 (0.869)**	**0.011 (0.627)**
apigenin-7-O-rutinoside (isorhoifolin)	**<0.001 (0.943)**	**<0.001 (0.916)**	**<0.001 (0.769)**
naringenin	**0.082 (0.566)**	**0.005 (0.649)**	0.423 (0.257)
diosmetin	**<0.001 (0.908)**	**<0.001 (0.731)**	**0.050 (0.506)**

^†^ For within-subject effects, Greenhouse–Geisser-corrected *p*-values were reported throughout; the between-subject effect of cultivar needs no such correction. The *p*-values shown are raw; Benjamini–Hochberg-adjusted values are given in Supplementary Table S5. Values in parentheses represent partial eta-squared (ηp^2^). Statistically significant effects (*p* ≤ 0.05) are shown in bold.

### 3.6. Secoiridoids and Related Compounds

Sampling period significantly affected all four secoiridoids and related compounds (Table 6). The strongest temporal effect was observed for oleuroside (ηp^2^ = 0.927), followed by 11-methyloleoside (ηp^2^ = 0.728), oleuropein (ηp^2^ = 0.685), and secologanoside (ηp^2^ = 0.581). In contrast, cultivar effects were restricted to 11-methyloleoside (*p* = 0.025, ηp^2^ = 0.709), oleuroside (*p* = 0.008, ηp^2^ = 0.797), and oleuropein (*p* = 0.049, ηp^2^ = 0.635); the last of these was retained under neither Benjamini–Hochberg definition (*q* = 0.089 within the model term, *q* = 0.073 within the compound) and is therefore provisional.

**Figure 5:**
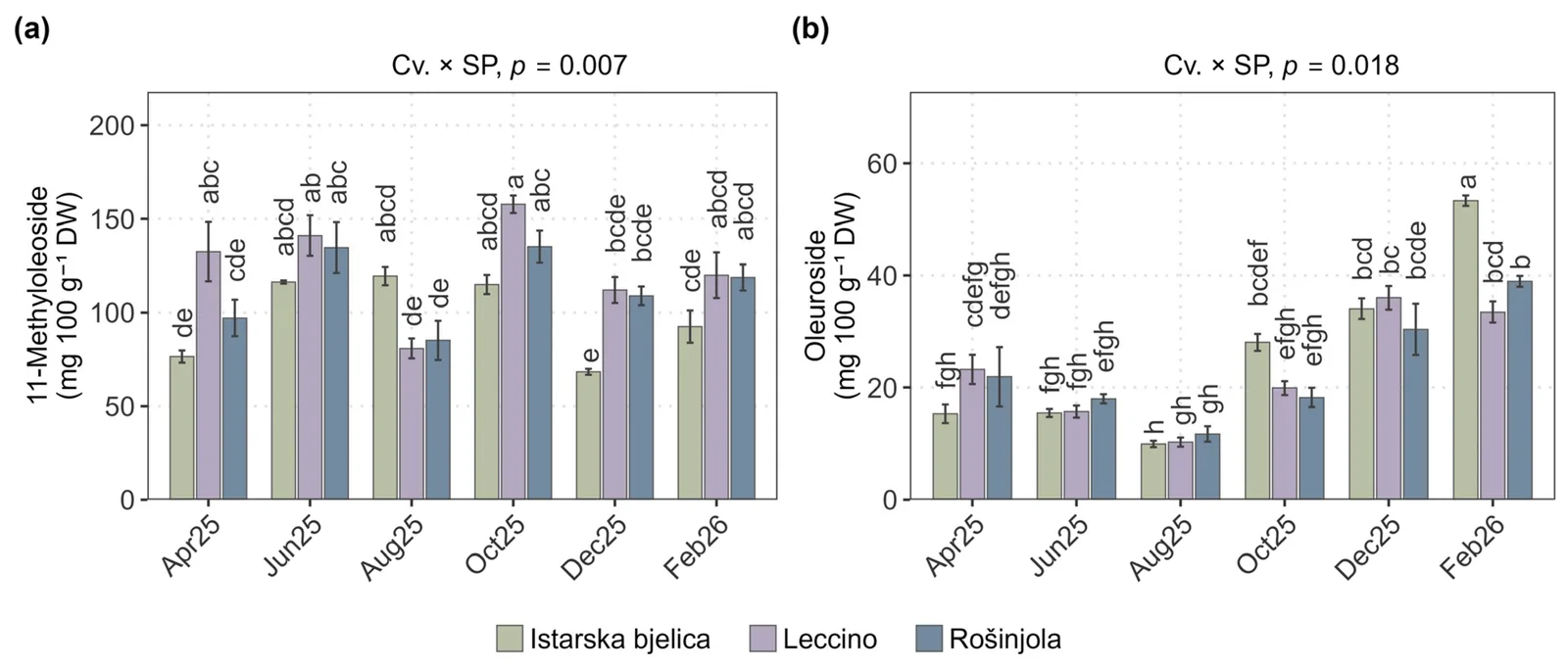
Temporal changes in secoiridoids and related compounds showing a significant cultivar × sampling period interaction, among three olive cultivars from April 2025 to February 2026. (**a**) 11-Methyloleoside, (**b**) oleuroside. Values are presented as means ± standard errors (n = 3). For significant cultivar × sampling period interactions, cultivars were compared within sampling periods using Tukey’s test and sampling periods within cultivars using Bonferroni-adjusted comparisons (*p* ≤ 0.05); different letters indicate significant simple-effect differences. The *p*-value above each panel is the Greenhouse–Geisser-corrected interaction term.

These two compounds also showed significant cultivar × sampling period interactions, with effect sizes of ηp^2^ = 0.659 for 11-methyloleoside (*p* = 0.006) and ηp^2^ = 0.648 for oleuroside (*p* = 0.009). The 11-methyloleoside profiles fluctuated markedly across the sampling period, reaching their highest concentrations in October in ‘Leccino’ and ‘Rošinjola’, while the increase in ‘Istarska bjelica’ was less pronounced (Figure 5a). Oleuroside concentrations decreased from April to August and subsequently increased in all three cultivars. This increase was most pronounced in ‘Istarska bjelica’, which reached the highest concentration in February 2026 (Figure 5b).

**Table 6:** Effects of cultivar, sampling period, and their interaction on secoiridoids and related compounds in olive samples.

Compound	Cultivar *p* (ηp^2^)	Sampling Period *p* ^†^ (ηp^2^)	Cultivar × Sampling Period *p* ^†^ (ηp^2^)
secologanoside	0.655 (0.132)	**0.003 (0.581)**	0.094 (0.443)
11-methyloleoside	**0.025 (0.709)**	**<0.001 (0.728)**	**0.005 (0.659)**
oleuropein	**0.049 (0.635)** ^‡^	**<0.001 (0.685)**	0.363 (0.282)
oleuroside	**0.008 (0.797)**	**<0.001 (0.927)**	**0.014 (0.648)**

^†^ For within-subject effects, Greenhouse–Geisser-corrected *p*-values were reported throughout; the between-subject effect of cultivar needs no such correction. The *p*-values shown are raw; Benjamini–Hochberg-adjusted values are given in Supplementary Table S5. Values in parentheses represent partial eta-squared (ηp^2^). Statistically significant effects (*p* ≤ 0.05) are shown in bold; ^‡^ raw *p* ≤ 0.05 but not retained under either Benjamini–Hochberg definition.

### 3.7. Principal Component Analysis of Chemical Profiles According to Cultivar and Sampling Period

**Figure 6:**
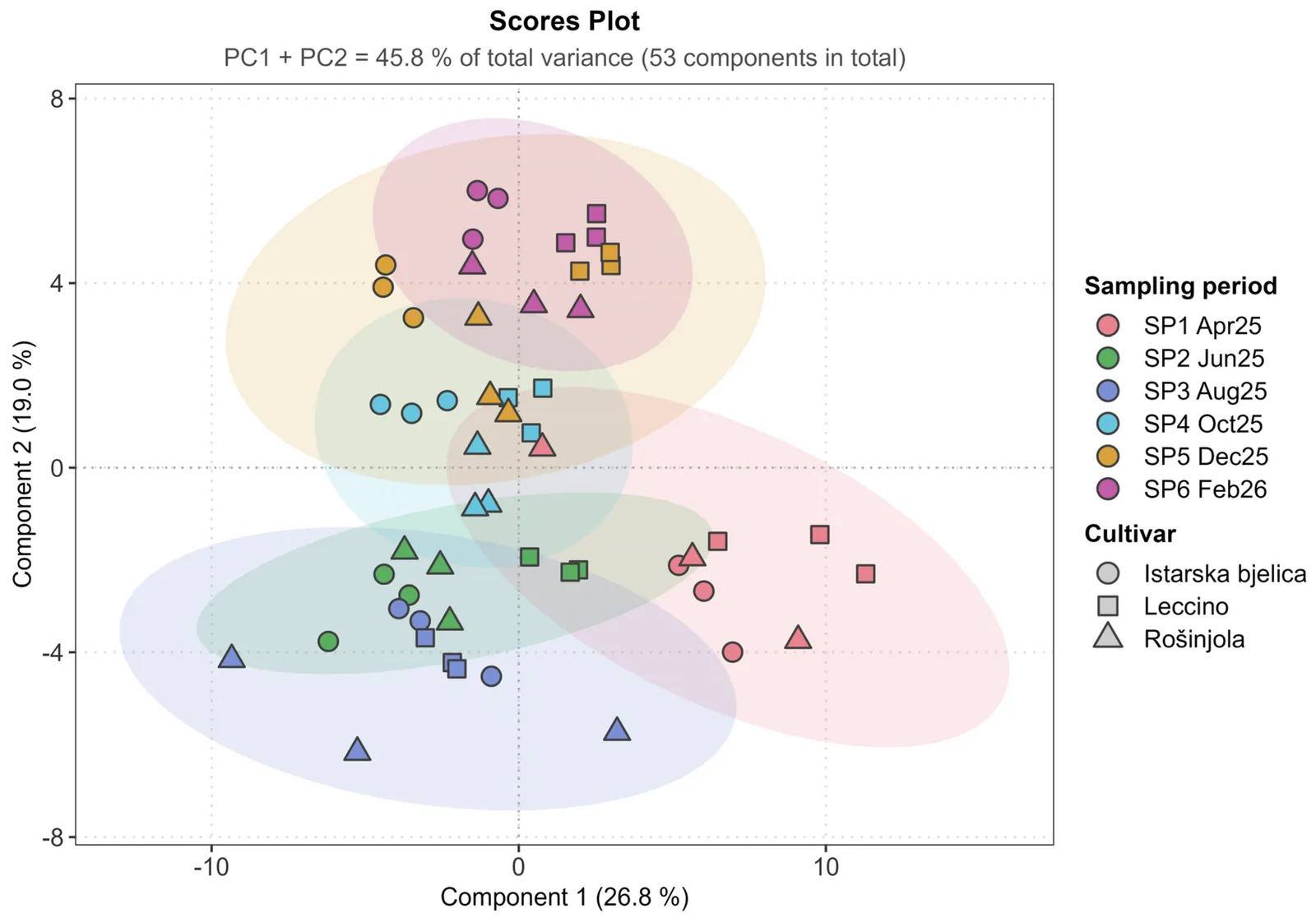
Principal component analysis (PCA) score plot of the metabolic profiles of olive samples. Symbol colour indicates the sampling period (April 2025–February 2026) and symbol shape indicates the cultivar ‘Istarska bjelica’, ‘Leccino’ and ‘Rošinjola’; shaded areas are 95% confidence ellipses drawn per sampling period.

PCA was performed on the log-transformed dataset comprising 64 variables to examine overall patterns in the chemical profiles of the olive samples. PC1 explained 26.81% of the total variance, while PC2 accounted for an additional 18.96%, resulting in a cumulative explained variance of 45.77% for the first two components (Figure 6). PC3 and PC4 explained an additional 10.00% and 7.37% of the variance, respectively, increasing the cumulative explained variance to 55.77% for the first three components and 63.14% for the first four. The PC1–PC2 plane was used to visualise the main patterns in sample distribution. When the PCA scores were examined according to cultivar (Figure 6), considerable overlap among ‘Leccino’, ‘Rošinjola’, and ‘Istarska bjelica’ was observed in the central region of the PC1–PC2 space. Nevertheless, cultivar-related tendencies were apparent. ‘Leccino’ samples were predominantly distributed towards positive PC1 scores, with a substantial proportion extending towards negative PC2 values. ‘Rošinjola’ showed the broadest distribution along PC1, including several samples with markedly negative PC1 scores, while others occupied positive PC1 and PC2 regions. ‘Istarska bjelica’ was distributed mainly between negative and moderately positive PC1 scores, with an extension towards negative PC2 values. Overall, the three cultivars did not form clearly separated groups, although differences in their distributions were evident.

A clearer pattern emerged when the same PCA scores were examined according to sampling period (Figure 6). Samples collected in April 2025 were predominantly positioned in the positive PC1 region and generally showed positive PC2 scores, whereas June samples occupied mainly the upper central region of the plot. August samples displayed the widest distribution towards negative PC1 scores, including the most negative PC1 observations in the dataset. October samples were concentrated closer to the centre of the PCA space, primarily around slightly negative to positive PC1 scores and moderately negative PC2 scores. A pronounced shift towards negative PC2 scores was evident in December 2025 and February 2026. These two later sampling periods showed considerable overlap, although February samples tended to occupy somewhat more positive PC1 positions. Overall, the progression of samples across the PC1–PC2 space from April 2025 to February 2026 revealed a more distinct temporal pattern than the separation according to cultivar.

The variable loadings provided further information on the metabolites contributing to the observed distribution of samples (Supplementary Table S6). Several amino acids showed strong positive loadings on PC1, including threonine (PC1 = 0.952), serine (0.944), aspartic acid (0.899), leucine (0.895), and isoleucine (0.888). 2-Aminobutyric acid and tyrosine also exhibited strong positive PC1 loadings (0.848 and 0.846, respectively). Samples positioned towards the positive side of PC1 were therefore aligned with comparatively greater contributions from this group of variables.

PC2 reflected a different compositional gradient. Dihydroquercetin (taxifolin) showed the strongest positive PC2 loading among the labelled variables (0.841), followed by 3,4-dihydroxybenzoic acid (protocatechuic acid; 0.830), quercetin-4′-O-glucoside (spiraeoside; 0.767), dimethylglycine (0.682), and choline (0.659). Conversely, oleuroside (PC2 = −0.915), glutamine (−0.856), and naringenin-7-O-glucoside (−0.819) were associated with the negative direction of PC2. Glutamic acid (−0.592) and 4-aminobutyric acid (−0.702) also had negative PC2 loadings, although their contribution to sample differentiation was also associated with their positive PC1 loadings.

**Figure 7:**
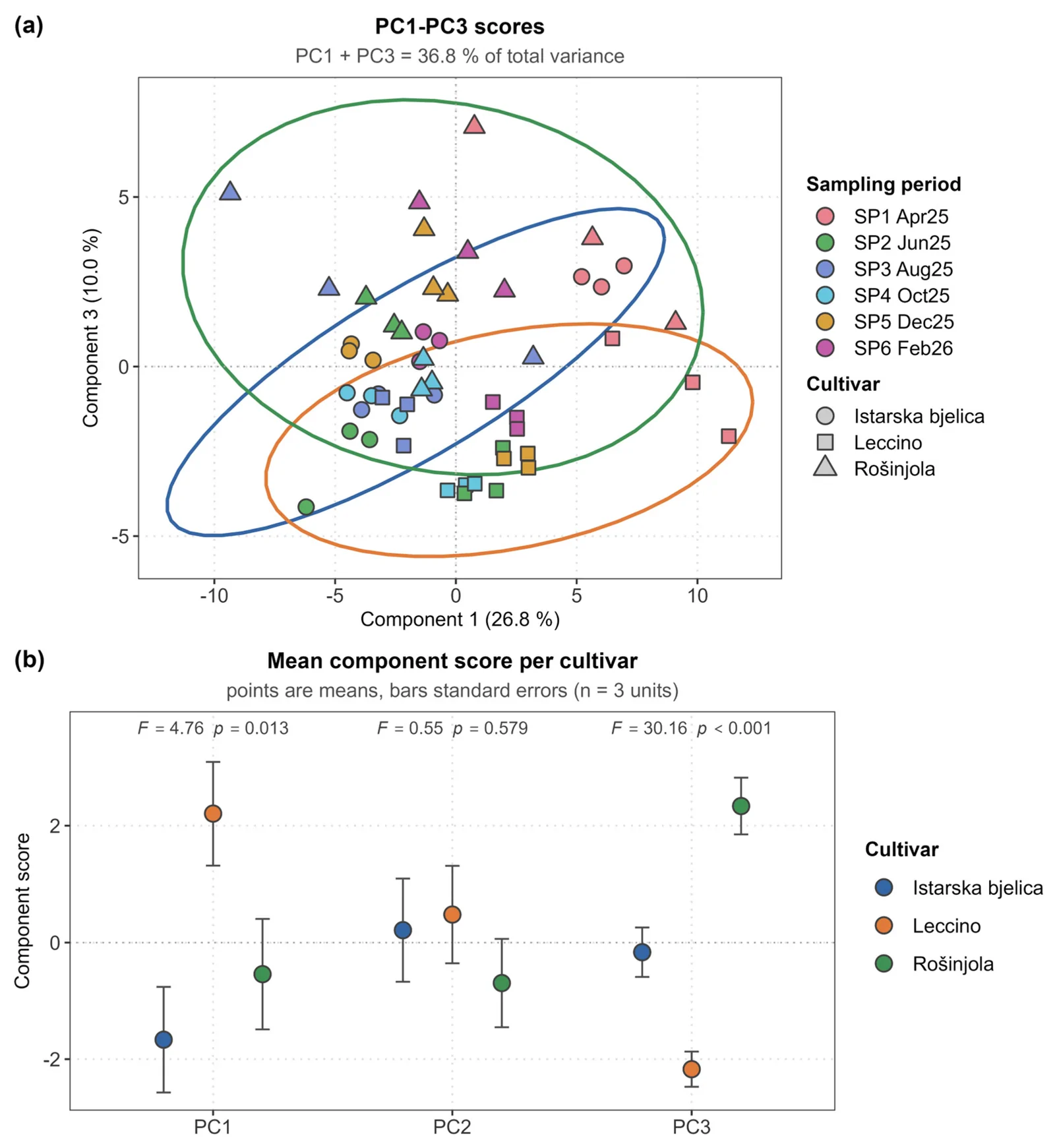
Third principal component of the metabolic profiles of olive samples, from the same principal component analysis as Figure 6 (n = 54 samples). (**a**) PC1–PC3 score plot: symbol colour indicates the sampling period and symbol shape indicates the cultivar; outlines are 95% confidence ellipses drawn per cultivar, coloured as in panel (**b**). (**b**) Mean component score per cultivar for PC1–PC3, with standard errors; one-way comparisons of component scores by cultivar gave F = 30.16, *p* < 0.001 for PC3; F = 4.76, *p* = 0.013 for PC1, and F = 0.55, *p* = 0.579 for PC2. PC3 accounts for 10.00% of the total variance.

The loading structure corresponded to the temporal distribution observed in the score plot. Samples from the earlier sampling periods, particularly April, occupied predominantly positive PC1 and PC2 regions and were aligned with variables loading positively on these components. In contrast, the shift in December 2025 and February 2026 samples towards negative PC2 corresponded to the direction of oleuroside, glutamine, and naringenin-7-O-glucoside. The extension of August samples towards negative PC1 was opposite to the direction of the amino acids and related compounds characterised by high positive PC1 loadings. Complete PC1-PC3 loadings for all 64 variables included in the analysis are provided in Supplementary Table S6. Because the PC1–PC2 plane captured 45.77% of the variance, the third component was examined as well (Figure 7). PC3 explained a further 10.00% and, unlike PC1 and PC2, separated the cultivars, with ‘Rošinjola’ and ‘Leccino’ at opposite extremes (F = 30.16, *p* < 0.001 against F = 4.76, *p* = 0.013 for PC1 and F = 0.55, *p* = 0.579 for PC2).

### 3.8. Relationships Among Individual Metabolites: Spearman Correlation Analysis

Spearman’s rank correlation analysis revealed numerous strong associations among the analysed metabolites, with the highest positive correlations occurring predominantly among amino acids (Supplementary Table S7). The strongest correlation was observed between isoleucine and leucine (ρ = 0.966), followed by serine and threonine (ρ = 0.944), valine and isoleucine (ρ = 0.925), choline and dimethylglycine (ρ = 0.924), and valine and leucine (ρ = 0.923). Other strong positive correlations included alanine and glutamic acid (ρ = 0.919), isoleucine and 2-aminobutyric acid (ρ = 0.908), serine and leucine (ρ = 0.905), and threonine and valine (ρ = 0.903), demonstrating extensive co-variation within the amino acid profile. Because the same experimental units were sampled repeatedly, these correlations are exploratory and do not by themselves demonstrate co-regulation: compounds that follow similar seasonal trajectories yield a high coefficient whether or not they are metabolically linked.

Strong positive correlations were also found among phenolic compounds and related metabolites. Naringenin-7-O-glucoside was strongly correlated with oleuroside (ρ = 0.866), while luteolin and apigenin were positively correlated (ρ = 0.814). Dihydroquercetin (taxifolin) showed a strong association with quercetin-4′-O-glucoside (spiraeoside) (ρ = 0.883), as did chlorogenic acid with luteolin-7-O-glucoside (ρ = 0.758). Strong correlations were therefore not confined to compounds within the same chemical group but were also observed between metabolites from different groups.

Negative associations were much less frequent. Four correlations met the predefined threshold for a strong negative association (ρ ≤ −0.70). Three involved oleuroside: with 3,4-dihydroxybenzoic acid (protocatechuic acid) (ρ = −0.783), dihydroquercetin (taxifolin) (ρ = −0.753), and quercetin-4′-O-glucoside (spiraeoside) (ρ = −0.717); the fourth was between luteolin and luteolin-3,7-O-glucoside (ρ = −0.700). Overall, strong correlations were dominated by positive associations, particularly among amino acids. All metabolite pairs with |ρ| ≥ 0.70 are reported in Supplementary Table S7.

### 3.9. Supervised Classification of Metabolite Profiles: PLS-DA

**Figure 8:**
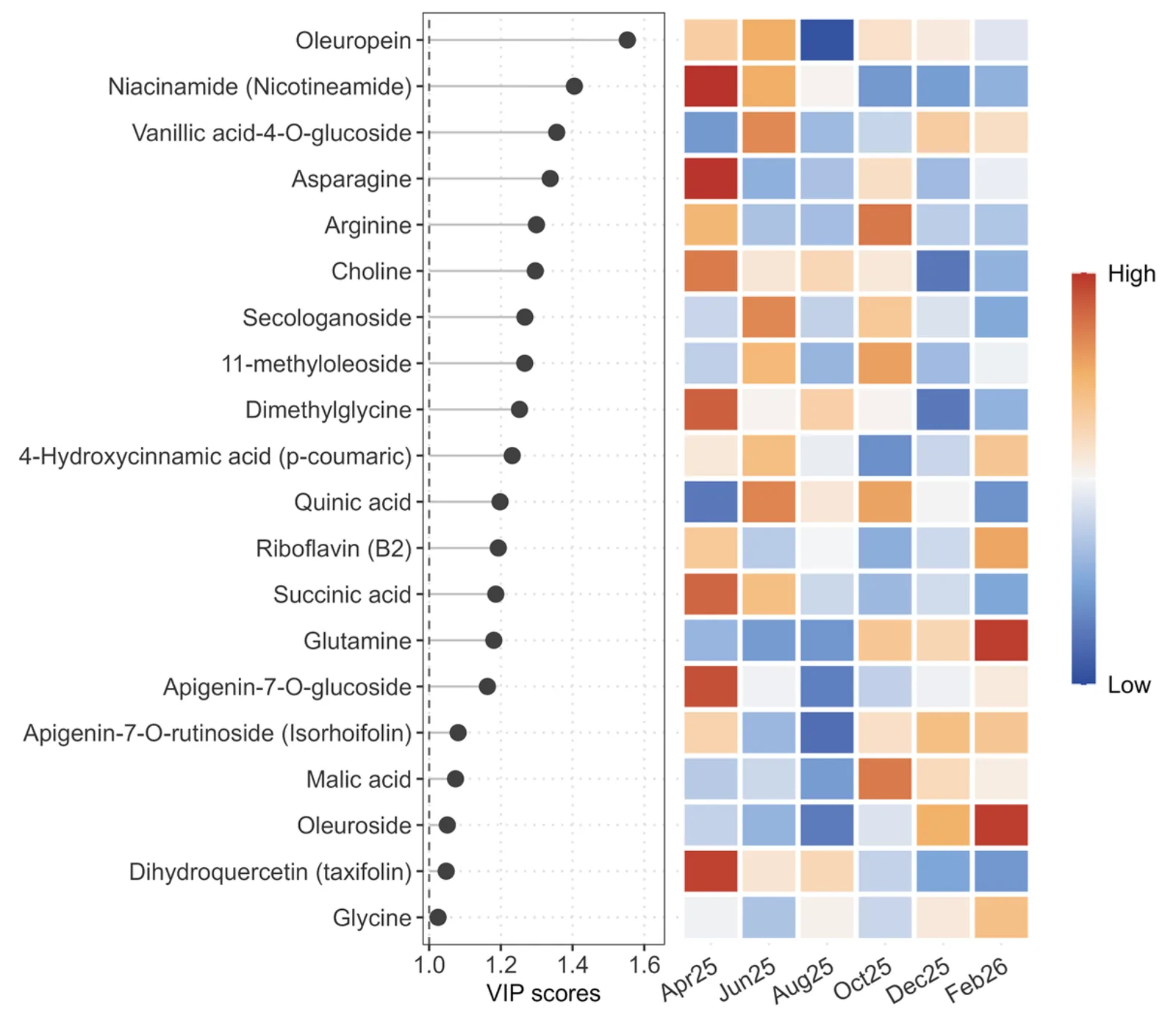
PLS-DA variable importance in projection (VIP) scores for the sampling period model. The 20 highest-ranking of the 28 metabolites with VIP > 1 are shown (28 of 64); the coloured panel indicates the group mean of each metabolite on the standardised scale, from low (blue) to high (red). Model: 8 components, leave-one-experimental-unit-out cross-validation accuracy 0.981, permutation *p* = 0.001.

**Figure 9:**
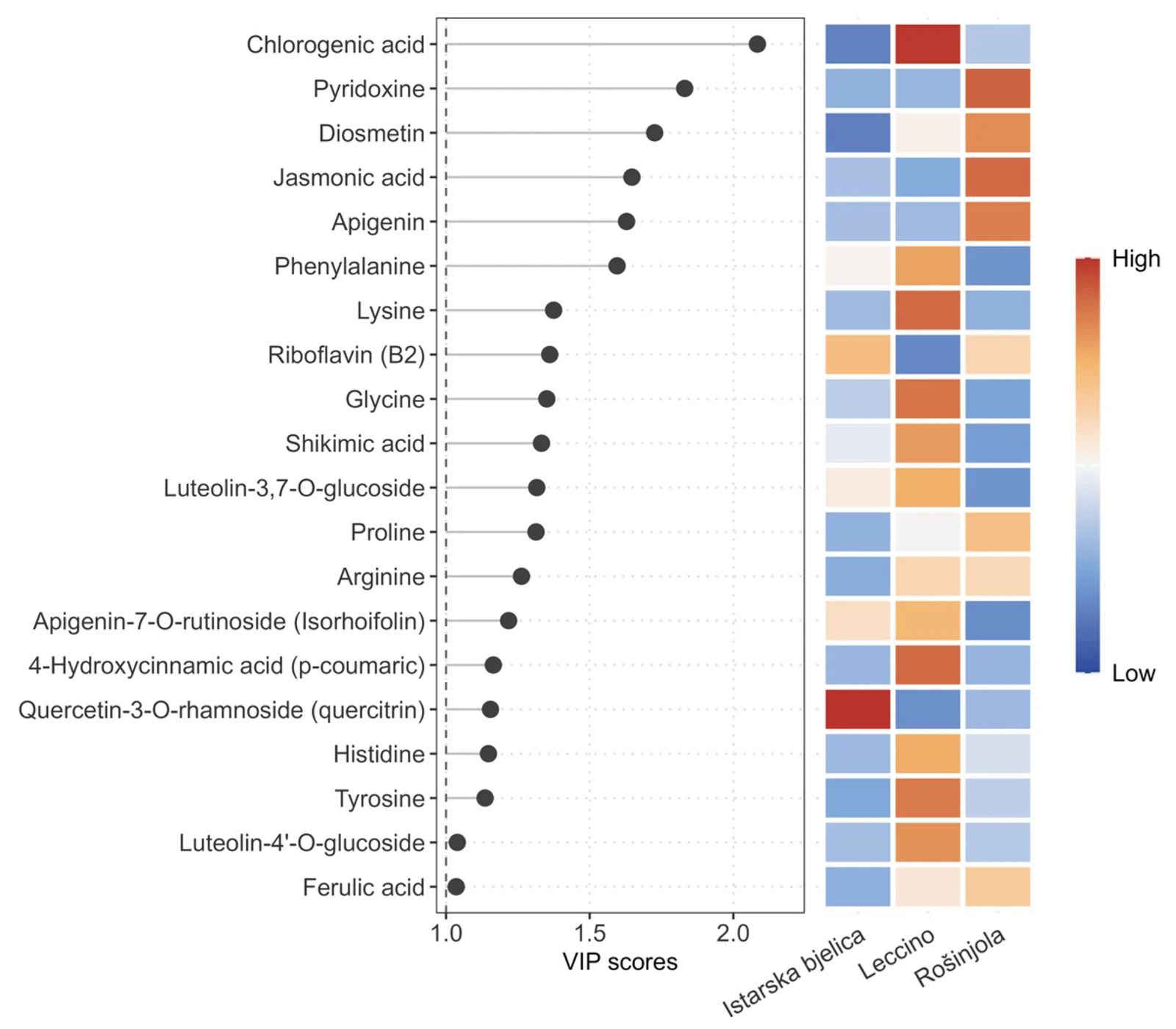
PLS-DA variable importance in projection (VIP) scores for the cultivar model. The 20 highest-ranking of the 21 metabolites with VIP > 1 are shown (21 of 64); the coloured panel indicates the group mean of each metabolite on the standardised scale, from low (blue) to high (red). Model: 4 components, leave-one-experimental-unit-out cross-validation accuracy 0.963, permutation *p* = 0.012.

Partial least squares discriminant analysis (PLS-DA) was used to evaluate if metabolite profiles could effectively classify samples based on sampling period and cultivar. Since the same experimental units were sampled multiple times, cross-validation involved leaving out one entire experimental unit at a time, and model significance was tested via permutation within experimental units and cultivar labels between whole units (999 and 249 permutations, respectively). The number of latent variables in PLS-DA is not bounded by the number of classes. For sampling period, accuracy increased up to eight components, where the model correctly classified 98.1% of samples in leave-one-experimental-unit-out cross-validation, well above the 16.7% chance level for six groups; permutation of period labels within experimental units confirmed the separation (*p* = 0.001). Twenty-eight of 64 metabolites had VIP > 1, with the highest values for oleuropein, niacinamide, vanillic acid-4-O-glucoside, asparagine, arginine, and choline (Figure 8). For cultivar, cross-validated accuracy reached its plateau at four components (96.3%; chance level 33.3%), and permutation of whole-unit cultivar labels validated the separation (*p* = 0.012); adding further components did not improve performance. With this model, 21 metabolites had VIP > 1, led by chlorogenic acid, pyridoxine, diosmetin, jasmonic acid, apigenin and phenylalanine (Figure 9; full VIP scores in Supplementary Table S6). Unlike the PCA score plot, where cultivars overlapped extensively, these results show cultivar differences exist but are spread across many metabolites rather than confined to the first two principal components.

### 3.10. Contribution of Cultivar and Sampling Period at the Whole-Metabolome Level

**Figure 10:**
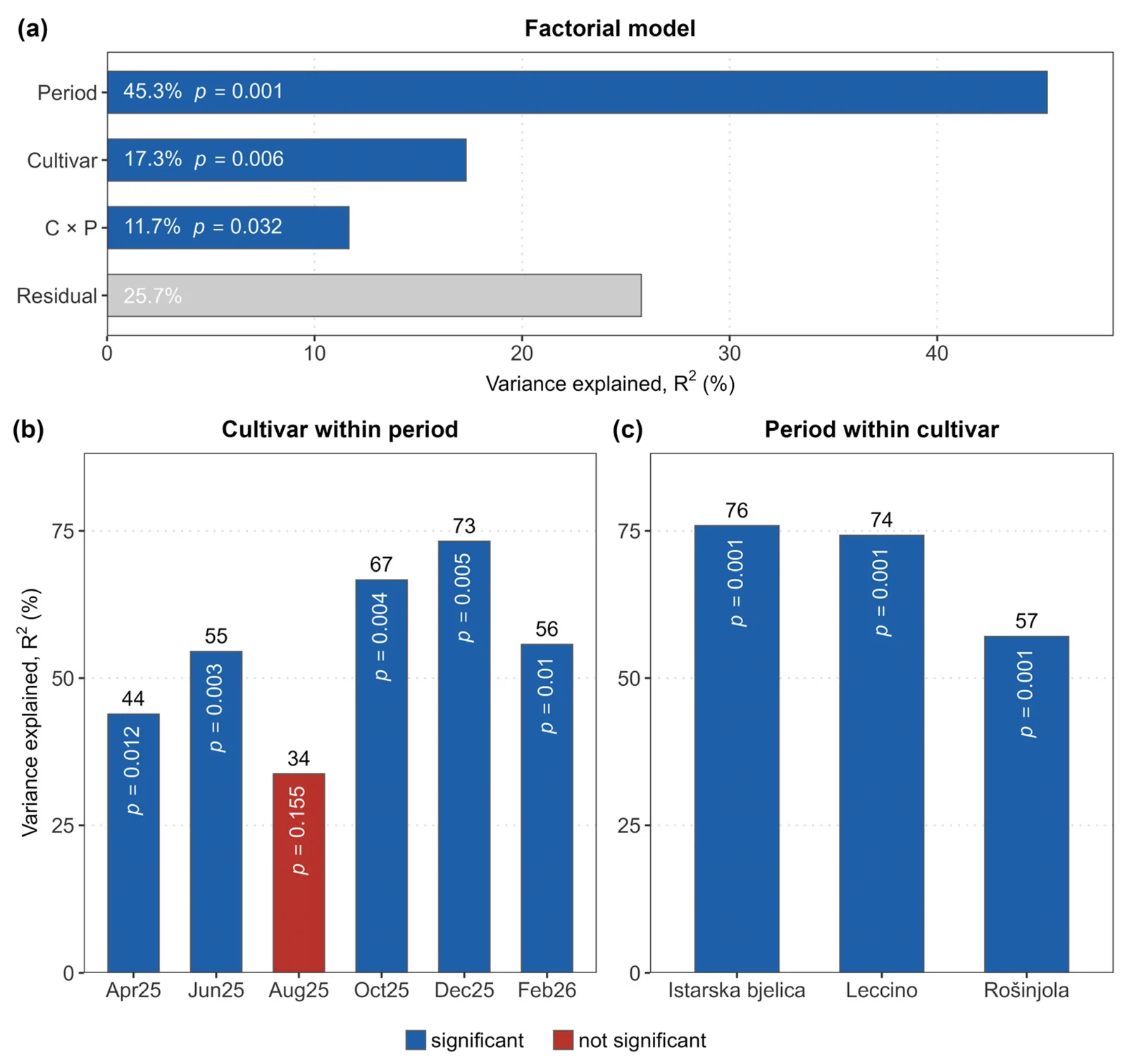
Contribution of sampling period and cultivar to variation in the olive leaf metabolome, assessed by PERMANOVA on the Euclidean distance matrix of the log-transformed, *z*-standardised concentrations of 64 metabolites (n = 54 samples). (**a**) Factorial model (sampling period + cultivar + sampling period × cultivar): variance explained (*R*^2^) by each term, computed sequentially by terms. (**b**) Cultivar effect within each sampling period and (c) sampling-period effect within each cultivar: variance explained (R^2^) with the permutation *p* above each bar (n = 9 per period and n = 18 per cultivar, respectively; cultivar labels permuted between whole units, period labels within units). All tests used 999 permutations. Panels (**b**,**c**) share a common *y* axis.

To quantify how much of the variation in the metabolome as a whole is attributable to each factor, PERMANOVA was applied to the Euclidean distance matrix of the log-transformed, z-standardised concentrations of all 64 metabolites. Sampling period accounted for 45.3% of the total variance (pseudo-F = 12.67, *p* = 0.001), and cultivar for 17.3% (pseudo-F = 12.10, *p* = 0.006); both factors were therefore significant, but the contribution of sampling period was 2.6-fold larger (Figure 10a). The interaction accounted for a further 11.7% (pseudo-*F* = 1.63, *p* = 0.032), leaving 25.7% residual; the four terms sum to 100%.

The separate permutation tests shown in Figure 10b,c were used to examine each factor within levels of the other factor. For sampling period, no permutation of period labels within experimental units reached the observed pseudo-F (highest permuted value 2.05 against 7.95 observed). For cultivar, three of the 999 permutations reproduced the observed pseudo-F, giving *p* = 0.004. Because only 1680 distinct assignments of the nine experimental units among the three cultivars are possible, identical permutations can occur. Ties were included in the *p*-value calculation to ensure a conservative and reproducible result. Multivariate dispersions were homogeneous for both factors (PERMDISP: sampling period *F* = 1.00, *p* = 0.418; cultivar *F* = 0.21, *p* = 0.839), so the PERMANOVA results can be interpreted as differences in centroid location rather than in within-group dispersion.

## 4. Discussion

The results showed that the sampling period influenced the olive leaf metabolic profile more than the cultivar. The cultivar × sampling period interaction was significant for 23 metabolites. Of the remaining 41 detected metabolites, 22 showed a significant effect of sampling period only, three showed a significant effect of cultivar only, and another 11 showed both main effects of cultivar and sampling period without a significant interaction. Five metabolites showed no effect of sampling or cultivar. In any case, the effect of sampling period was significant in 56 metabolites and cultivar in 35, and these totals include the metabolites with a significant interaction. Counting significant tests does not by itself quantify the relative weight of the two factors, so generalised eta-squared was compared across all 64 metabolites: the median was 0.710 for sampling period and 0.322 for cultivar, a 2.2-fold difference that was highly consistent (sampling period exceeded cultivar in 50 of 64 metabolites; Wilcoxon signed-rank *p* = 6.3 × 10^−7^; per-metabolite values in Supplementary Table S5). At the multivariate level, the same ordering held: PERMANOVA attributed 45.3% of the total variance to sampling period and 17.3% to cultivar, with a further 11.7% to their interaction (Section 3.10, Figure 10). Although sampling period significantly affected the overall metabolite profile of all three cultivars, the temporal separation was less pronounced in ‘Rošinjola’, suggesting a comparatively more stable metabolomic profile across the annual cycle. Multivariate dispersions were homogeneous for both factors (PERMDISP *p* = 0.418 for sampling period and *p* = 0.839 for cultivar), so these differences in metabolome composition indicate that the significant PERMANOVA effects were not accompanied by detectable differences in within-group dispersion. The interaction was itself asymmetric: cultivar separated the profiles in five of the six sampling periods but not in August, whereas sampling period was significant within every cultivar. The seasonal signal was therefore consistent across all three cultivars, while cultivar differentiation appears through the annual cycle. This is also the case in unsupervised multivariate analyses. The first two principal components of the analysis accounted for 26.81% and 18.96% of total variance, respectively. In general, the samples were ordered along these components according to sampling period, with cultivars overlapping, and no principal component separated the cultivars.

Because each of the 64 metabolites was tested separately, the *p*-values reported in Table 1, Table 2, Table 3, Table 4, Table 5 and Table 6 are nominal. The Benjamini–Hochberg correction was therefore applied post hoc, with the family defined in two ways—within each model term (64 tests per term) and within each metabolite (3 tests per metabolite)—and is reported alongside the nominal values in Supplementary Table S5. Of 114 effects significant at raw *p* ≤ 0.05, 94 were retained under both definitions and three under neither; the latter are marked in Table 1, Table 3 and Table 6. The remaining 17 were retained under one definition only and are therefore scheme-dependent; they are significant but unmarked in Table 1, Table 2, Table 3, Table 4, Table 5 and Table 6. The sampling period was unaffected (56 of 64 under either definition), whereas the interaction count fell from 23 to 10 within model terms. Approximately three significant results would be expected by chance at α = 0.05; the observed counts exceed this by 17.5-fold for sampling period and 10.9-fold for cultivar, and the conclusion that sampling period outweighs cultivar rests on the relative magnitude of the effects rather than on these counts. Individual effects with *p* close to 0.05—in particular the cultivar effects on oleuropein (*p* = 0.049; *q* = 0.089), quinic acid (*p* = 0.048; *q* = 0.089) and asparagine (*p* = 0.041; *q* = 0.079), the effects retained under neither definition, and the eight interactions with *p* above 0.028—should therefore be regarded as provisional and treated as candidates for targeted confirmation rather than as established cultivar differences. Summed concentrations by compound group are given in Supplementary Table S8.

Although cultivars are not separated in these unsupervised displays, cultivar differences are nevertheless present. The PLS-DA model classified samples by cultivar with 96.3% cross-validated accuracy using four components (permutation *p* = 0.012), with 21 metabolites having VIP > 1 (Supplementary Table S6). Because only nine experimental units were available, three per cultivar, this accuracy describes separation within the present dataset and is not evidence of predictive performance beyond it. Therefore, cultivar differences do exist at the multivariate level; however, they are distributed across a larger number of metabolites and are not concentrated in the first two principal components. The third component shows this directly: unlike PC1 and PC2 it separates the cultivars, F = 30.16, *p* < 0.001, so the signal is present in the unsupervised ordination but carried by a lower-variance component. This is a significant finding from a methodological perspective, since concluding from a PCA score plot that cultivars do not differ may be misleading.

Primary metabolites in olive leaves have shown seasonal patterns. The total amount of amino acids and other N compounds reached the highest level in spring. Among them, asparagine (Asn) was the most abundant free amino acid detected, suggesting its pivotal role as an N-containing compound translocated in olive leaves during spring growth. As reported by Malaguti et al. [38], most of the labelled N was found in Asn, glutamine (Gln), glutamic acid (Glu), and aspartic acid (Asp), indicating these metabolites were translocated due to remobilisation in apple trees. Furthermore, other researchers noted that remobilisation to young tissues in broadleaf species like olive is most likely linked to storage in old leaves and stems [39]. Among all amino acids, arginine (Arg) has also been identified as the main amino acid linked to N mobilisation in forest trees, connected to Gln and Asn metabolism [40]. In our research, these seasonal fluctuations in nitrogen metabolism are evident across all cultivars for Asn and Asp, but Gln, Glu, and Arg show distinct variation between cultivars due to C x ST interaction (cultivar × sampling period interaction). Seasonality of Asn and Arg has been confirmed by the highest VIP scores in our research (Figure 8). Interestingly, proline is often described as a plant stress-response indicator because it responds to low temperature, drought, and salinity [41,42,43]. In our data, it is highest in April, which corresponds with previously noted seasonal patterns of vegetative growth and N metabolism related to observed amino acid fluctuations rather than an adaptive environmental stress response [43]. Raza et al. [44] noted that proline is not only a stress metabolite; it also functions as a source of carbon and nitrogen, and it is also synthesised in unstressed environments.

The relative contributions of sampling period and cultivar, already evident at the level of primary nitrogen metabolism, also underlie the accumulation of secondary metabolites, although the weight of each factor differs markedly between compound classes and biosynthetic pathways. Olive leaf phenolic composition can also be modified by mineral nutrition, particularly through foliar nutrient application [3,4,45]. Among phenolic acids and flavonoids, the two downstream branches of the phenylpropanoid pathway, cultivar and sampling period again acted with different relative strength, consistent with the C x ST pattern observed for Asn, Gln, Glu and Arg (Table 4 and Table 5). Phenolic acids showed a heterogeneous, compound-specific response, with seven of the nine compounds varying significantly across sampling periods and only four showing a significant cultivar effect, whereas flavonoids showed pronounced effects of both cultivar and sampling period, acting as a secondary modulator [46]. This dual regulation is consistent with the branching role of the phenylpropanoid pathway, whose activity is largely cultivar-dependent while remaining responsive to seasonal environmental variation, consistent with previous observations [47,48]. The same cultivar-dependent seasonality of leaf phenolics has been reported for Istrian cultivars under both foliar treatment and repeated sampling [3,32].

Chlorogenic acid was the most discriminating phenolic acid, combining the strongest cultivar effect recorded in the study (ηp^2^ = 0.988) with a significant sampling-period effect and a pronounced cultivar x sampling-period interaction (ηp^2^ = 0.814; Figure 3a); ‘Leccino’ accumulated consistently higher concentrations than ‘Istarska bjelica’ and ‘Rošinjola’, peaking in April (56.62 mg 100 g^−1^ DW; Table S4). This genotypic differentiation is consistent with the findings of Palmeri et al. [49] and Altomare et al. [50]. However, Khelouf et al. [51] reported that olive leaves are generally dominated by oleuropein, hydroxytyrosol and verbascoside, chlorogenic acid remaining a comparatively minor but strongly cultivar-dependent marker. Protocatechuic acid showed a strong seasonal response (peak in August, trough in February), and hydroxytyrosol followed a comparable seasonal decline (7.17 to 2.29 mg 100 g^−1^ DW) without any cultivar effect (*p* = 0.180), indicating that its accumulation was primarily associated with seasonal variation rather than cultivar, consistent with Khelouf et al. [51]. Vanillic acid-4-O-glucoside, in contrast, was significantly affected by cultivar, sampling period, and their interaction (Figure 3b), with shifting cultivar rankings over time that may reflect cultivar-specific acclimation through glycosylation. The cultivar dependence of oleuropein reported here is also supported by an independent dataset from a different growing region. In six cultivars from central Dalmatia sampled at harvest (October) and pruning (March), cultivar rather than sampling period was the dominant source of variation for oleuropein and total phenols, whereas sampling period dominated for hydroxytyrosol [52]. Only ‘Leccino’ is common to both studies, so the comparison is between designs rather than between the same genotypes: two sampling dates set at the extremes of the annual cycle maximise the cultivar contrast, whereas the six-period design used here resolves the seasonal trajectory and shows that oleuropein passes through a sharp August minimum. The relative weight of the two factors therefore depends on how the annual cycle is sampled, which is consistent with the provisional status of the oleuropein cultivar effect noted above.

Of the four simple phenolic acids quantified, only p-coumaric acid and ferulic acid showed clear responses; caffeic acid and vanillin remained essentially stable. p-Coumaric acid was the only one significantly affected by cultivar, ‘Leccino’ accumulating almost twice the concentration of ‘Istarska bjelica’ and ‘Rošinjola’; as the precursor of p-coumaroyl-CoA, a branch point feeding flavonoid, lignin and lignan biosynthesis [53], this cultivar-dependent variation may partly underlie the flavonoid patterns described below. Seasonally, it followed a bimodal pattern (maxima in June and February), consistent with the cold-season increase in antioxidant phenolics reported by Blasi et al. [54]; ferulic acid rose sharply from December, peaking in February, a pattern consistent with previous reports describing the involvement of ferulic acid in antioxidant defence and lignification [55]. None of the four simple phenolic acids showed a significant cultivar x sampling-period interaction, indicating that cultivar mainly sets baseline concentrations rather than temporal dynamics, a pattern also reported across olive phenolics generally [56,57,58].

Flavonoids showed a markedly different balance. Cultivar significantly affected 11 of the 16 flavonoids and flavonoid glycosides quantified, with very large effect sizes (luteolin-7-O-glucoside, ηp^2^ = 0.962; isorhoifolin, ηp^2^ = 0.943; apigenin-7-O-glucoside, ηp^2^ = 0.942), while sampling period was significant for 14 compounds, most strongly for naringenin-7-O-glucoside (ηp^2^ = 0.924), isorhoifolin and luteolin-3,7-O-glucoside. This cultivar dominance agrees with prior work on the same cultivar triad [31,59] and with broader surveys showing cultivar as a key determinant of secondary-metabolite diversity across olive cultivars, total phenolic content varying more than tenfold among 61 international cultivars [60], luteolin-4′-O-glucoside dominating across eighteen Portuguese cultivars [61], and verbascoside showing the greatest genotypic variation among nine Turkish cultivars [62]. This cultivar dependence is mechanistically consistent with cultivar-correlated expression of biosynthetic transcripts [12] and with the role of Family-1 UDP-glycosyltransferases in governing glycoside accumulation [63,64], although no transcript or enzyme-activity measurements were made in the present study.

Seasonally, non-targeted profiling of twelve olive-leaf cultivars similarly detected marked autumn–spring differences [27]. Significant cultivar x sampling-period interactions were found for seven flavonoids, apigenin-7-O-glucoside showing the strongest (*p* < 0.001, ηp^2^ = 0.777, Figure 4a) and isorhoifolin close behind (Figure 4b), together with diosmetin, luteolin-3,7-O-glucoside, luteolin-4′-O-glucoside, luteolin-7-O-glucoside and naringenin-7-O-glucoside (Figure 4c–g); apigenin narrowly missed significance (*p* = 0.058). Beyond their chemotaxonomic value, luteolin and luteolin-7-O-glucoside are of direct nutraceutical relevance, with anti-inflammatory and antioxidant activity via STAT3 inhibition and reduced endothelial reactive oxygen species [65,66].

Secoiridoids, the iridoid-derived compounds characteristic of the Oleaceae, exhibited a contrasting pattern: sampling period was the principal driver of accumulation, with cultivar effects restricted to 11-methyloleoside, oleuropein and oleuroside (Table 6). Effect sizes for sampling period were largest for oleuroside (ηp^2^ = 0.927), followed by 11-methyloleoside (ηp^2^ = 0.728), oleuropein and secologanoside, consistent with harvest season and phenological stage as main determinants of olive-leaf phenolic composition [49,67]. Secologanoside, the most upstream compound, was affected exclusively by sampling period, peaking in June and declining towards February, consistent with its proposed role as a common precursor of oleosidic secoiridoids [26,68,69]. 11-Methyloleoside, by contrast, showed significant effects of cultivar, sampling period and their interaction, with ‘Leccino’ and ‘Rošinjola’ reaching an autumn maximum while ‘Istarska bjelica’ showed a flatter profile, consistent with its proposed role as an intermediate in oleuropein biosynthesis [26,70].

Oleuropein, the predominant secoiridoid in olive leaves, showed significant effects of both cultivar and sampling period without interaction, remaining high through spring and early summer before a sharp August decline, ‘Rošinjola’ showing the highest and ‘Istarska bjelica’ the lowest concentrations; this trajectory agrees with previous reports of peak oleuropein during flowering and active growth [49,71] and with declines from spring to summer or during fruit development reported elsewhere [67,72], underlining oleuropein’s status as the most abundant and biologically important olive secoiridoid [26]. Because the cultivar effect on oleuropein was retained under neither Benjamini–Hochberg definition (*p* = 0.049, *q* = 0.089; ‡ in Table 6), the ranking among cultivars is provisional, whereas the seasonal course is unambiguous (*p* < 0.001, *q* < 0.001). Oleuroside showed the strongest seasonal response overall and a significant cultivar x sampling-period interaction, decreasing from spring to summer and peaking in February, most markedly in ‘Istarska bjelica’, which paradoxically showed the lowest 11-methyloleoside, suggesting cultivar-specific partitioning of flux between secoiridoid forms [26]. Separately, oleuroside also showed strong negative correlations with protocatechuic acid and with the flavonoids taxifolin and spiraeoside (*p* ≤ −0.70), which is consistent with, but does not establish, a trade-off between oleuroside accumulation and other phenolic branches. The two principal cultivar-dependent phenolic markers identified above were themselves positively correlated (chlorogenic acid with luteolin-7-O-glucoside, *p* = 0.758), indicating that the two compounds follow similar seasonal trajectories; because the same experimental units were sampled repeatedly, such correlations are exploratory and do not by themselves demonstrate co-regulation.

These seasonal profiles may suggest a reorganisation of secoiridoid metabolism across the annual cycle, consistent with the complex, multi-enzyme biosynthetic network linking iridoid- and secoiridoid-derived intermediates, including iridoid synthase (ISY) as a key step in the oleuropein monoterpenoid scaffold [26,73], and with similar seasonal modifications reported elsewhere [49,67]. Practically, spring and early summer favoured secologanoside and oleuropein accumulation, autumn and winter favoured oleuroside, and ‘Istarska bjelica’ appears most promising for oleuroside recovery and ‘Leccino’ and ‘Rošinjola’ for 11-methyloleoside; the corresponding ranking for oleuropein rests on a cultivar effect that did not survive correction and would need confirmation before it guides cultivar choice. The prediction that cultivar differences would be more pronounced in both the flavonoid and the secoiridoid group was therefore supported only for flavonoids: cultivar was significant for 11 of 16 flavonoids and all 11 survived Benjamini–Hochberg correction, with generalised η^2^ for cultivar and sampling period almost equal (medians 0.678 and 0.724), whereas among the four secoiridoids cultivar reached significance for three but only one survived correction, and generalised η^2^ for sampling period exceeded that for cultivar 3.5-fold (medians 0.646 against 0.187).

Taken together, the three secondary-metabolite classes examined here can be positioned along a cultivar-environment continuum gradient already visible in primary nitrogen metabolism: flavonoids showed strong cultivar dependence superimposed on pronounced seasonal variation (ηp^2^ > 0.9 for several compounds), phenolic acids occupy an intermediate position in which cultivar fixes baseline concentrations while season modulates their timing, and secoiridoids were more strongly influenced by sampling period, with sampling period explaining most of the variance and cultivar effects confined to a specific subset of compounds. This distribution of effects may be consistent with the known regulation of these metabolic pathways: phenolic acids and flavonoids share a phenylpropanoid pathway whose activity is strongly influenced by genetic background, whereas secoiridoids derive from the iridoid pathway, which showed greater sensitivity to seasonal variation [73]. The recurring cultivar x sampling-period interactions across both pathways indicate that cultivars differ in their metabolic responses to seasonal changes, providing a practical basis for jointly optimising cultivar choice and harvest timing when targeting specific bioactive compounds for nutraceutical, pharmaceutical or food applications.

## 5. Conclusions

During the single annual cycle observed here, the sampling time had a stronger influence on the olive leaf metabolome than the cultivar itself: 56 out of 64 metabolites changed significantly depending on the sampling time, while 35 metabolites showed significant differences among the cultivars. Furthermore, the interaction between cultivar and sampling time was significant for 23 metabolites. Beyond these counts, effect sizes placed sampling time well ahead of cultivar: the median generalised eta-squared was 0.710 against 0.322, and PERMANOVA attributed 45.3% of the total metabolome variance to sampling time against 17.3% to cultivar. At the whole-metabolome level, sampling period significantly affected all three cultivars, whereas cultivar differentiation was not evident at every sampling period. Unsupervised PCA primarily grouped the olive leaf samples by season rather than cultivar. Although differences among cultivars were observed, they related to a larger number of metabolites rather than just a few dominant compounds. In contrast, supervised PLS-DA clearly differentiated cultivars, indicating that cultivar-related variation was distributed across multiple metabolites and was not fully captured by the first principal components.

The summed concentration of all 64 metabolites did not differ among cultivars, although three of the six compound classes did, suggesting that cultivars differ in metabolome composition rather than in overall metabolite quantity. These results emphasise the importance of sampling when comparing cultivars or analysing leaves, as the sampling time can alter the relative ranking of cultivars based on the content of individual compounds. The relative importance of cultivar and sampling period differed among metabolite classes, with stronger cultivar-related differentiation in flavonoids and a predominantly seasonal pattern in secoiridoids.

For the valorisation of olive leaves, it is also important to consider target compounds: the total secoiridoid content is highest in June and then decreases to its minimum in August, while free amino acids are most abundant in April; June and April are therefore the indicated harvest months for the two classes. Cultivar differences were therefore distributed across multiple metabolites, highlighting the need for standardised sampling dates when comparing cultivars or evaluating olive leaves as a raw material, under comparable conditions. Three limitations frame these conclusions. The orchard, the growing season and the three cultivars were single, so the seasonal trajectories described here are those of one site and one year and require confirmation elsewhere. Nine experimental units support the multivariate models, which constrains how far the classification accuracies can be extrapolated. The correlations among metabolites rest on repeated sampling of the same units and are therefore exploratory. Future research should further investigate the relationship between seasonal changes in metabolites, mineral content of the leaves, and oil quality across different cultivars within the same orchard or under similar agroecological conditions.

## Data Availability

The data supporting our findings and analyses are included in the article itself. Readers can access this data by referring to the article.

## Supplementary Materials

The following supporting information can be downloaded at: https://www.mdpi.com/article/10.3390/plants15213264/s1. Table S1: Analytical characteristics and data completeness of all 82 quantified metabolites; Table S2: Cultivar means ± standard error (mg 100 g^−1^ DW) with post hoc letters, for all 64 metabolites; Table S3: Sampling-period means ± standard error (mg 100 g^−1^ DW) with post hoc letters, for all 64 metabolites; Table S4: Cell means ± standard error (mg 100 g^−1^ DW) with simple-effect letters for the metabolites with a significant cultivar × sampling period interaction; Table S5: Effects of cultivar (Cv.), sampling period (SP) and their interaction on all 64 metabolites, with effect sizes, Benjamini–Hochberg-adjusted *p*-values and assumption checks; Table S6: Principal component loadings (PC1–PC3) and PLS-DA variable importance in projection (VIP) scores for the cultivar and sampling-period models, for all 64 metabolites; Table S7: Spearman rank correlations with |ρ| ≥ 0.70 among the 64 metabolites; Table S8: Summed concentrations by compound group (mg 100 g^−1^ DW), with repeated-measures ANOVA results.
